# Stylistic variation on the Donald Trump Twitter account: A linguistic analysis of tweets posted between 2009 and 2018

**DOI:** 10.1371/journal.pone.0222062

**Published:** 2019-09-25

**Authors:** Isobelle Clarke, Jack Grieve

**Affiliations:** Department of English Language and Linguistics, University of Birmingham, Birmingham, England, United Kingdom; University of Vermont, UNITED STATES

## Abstract

Twitter was an integral part of Donald Trump’s communication platform during his 2016 campaign. Although its topical content has been examined by researchers and the media, we know relatively little about the style of the language used on the account or how this style changed over time. In this study, we present the first detailed description of stylistic variation on the Trump Twitter account based on a multivariate analysis of grammatical co-occurrence patterns in tweets posted between 2009 and 2018. We identify four general patterns of stylistic variation, which we interpret as representing the degree of conversational, campaigning, engaged, and advisory discourse. We then track how the use of these four styles changed over time, focusing on the period around the campaign, showing that the style of tweets shifts systematically depending on the communicative goals of Trump and his team. Based on these results, we propose a series of hypotheses about how the Trump campaign used social media during the 2016 elections.

## Introduction

In comparison to other major candidates in the 2016 Republican primaries and the 2016 Presidential election, Donald Trump entered the race with a lack of support from politicians in his own party [1–3], the mainstream media [4], and large private donors [5], all of which have traditionally been seen as necessary for a successful campaign [3, 6]. Since Trump’s unprecedented success, many studies have attempted to explain how he overcame these obstacles to become the 45th President of the United States [7–12]. On Twitter, Trump gave at least some of the credit to his social media presence:

1 *How do you fight millions of dollars of fraudulent commercials pushing for crooked politicians*? *I will be using Facebook & Twitter*. *Watch*! (706675395811266560, 2016-03-06)
2 *I will be using Facebook and Twitter to expose dishonest lightweight Senator Marco Rubio*. *A record no-show in Senate*, *he is scamming Florida* (706829345143316480, 2016-03-07)

The claim that social media was integral to Trump’s campaign has been supported by several independent studies [13–19]. Perhaps most notably, it is estimated that coverage of Trump’s Twitter account (@realDonaldTrump) generated approximately 5 billion dollars of free media for the campaign [20].

Because of its importance to the campaign and the administration, the content of Trump’s Twitter account has been subject to intense scrutiny from both the media and academics. For example, broad topical patterns in the tweets sent during the general election from the accounts of Trump and Clinton have been compared through content analysis [18] and sentiment analysis [15], notably finding that Trump tended to be more positive than Clinton in the lead up to the election. Alternatively, adjectives in Trump’s Twitter have been found to be primarily negative and little variation was observed in the range of positive adjectives used [21]. Various studies have also fact-checked the tweets [22–23] and identified uses of logical fallacies [24].

Although the *content* of Trump’s Tweets has garnered considerable attention, the analysis of the *style* of Trump’s tweets–their form as opposed to their meaning–has been far more limited and has tended to focus on relatively superficial features, such as misspellings [25], insults [26–27], and non-standard grammar [28]. For example, we do not know the range of discursive styles and rhetorical strategies used on this account. We also do not know how the language of the account changed before, after, and during the campaign, especially because most previous studies focused on tweets sent during the election. Furthermore, almost all these studies have taken a top-down approach to data analysis, focusing on a small number of specific linguistic features judged to be of interest, rather than taking a bottom-up approach, letting data drive the analysis so as to produce a more complete description of the use of language on the account.

In addition to general analyses of the Trump Twitter account, the authorship of these tweets has also been examined to determine the extent to which Trump writes his own tweets [28–30]. Much of this research has been based on the assumption that Trump tweets from an Android phone as opposed to an iPhone [31]. For example, assuming that tweets sent from Android devices were written by Trump and tweets sent from iPhones were written by his staff, emotionally-charged and negative-sentiment words were found to be more common in tweets written by Trump [29]. Although we know Trump used an Android device during the campaign and that other members of the campaign likely had access to the account, it is problematic to infer authorship based on device, at the very least because other members of his campaign may have also used Android devices. Regardless, no matter how many authors have posted on the Trump Twitter account, and there may have been many, we believe its language as a whole is an important object of inquiry–a central part of the communication platform for Trump as a businessman, entertainer, candidate, and president.

The goal of this study is therefore to discover the most important general patterns of stylistic variation on the Trump Twitter account and to see how the style of language used on this account changed over time. We first identify and describe common styles of communication found in the complete corpus of tweets sent from the @realDonaldTrump Twitter account between 2009 and 2018 through a multivariate analysis of grammatical variation. We then track how the form of Trump’s tweets changed over time across these dimensions of stylistic variation, focusing especially on the 2016 presidential campaign, so as to better understand the communication strategy of Trump and his team. Our overarching aim is to produce an objective, impartial, and data-driven description of variation and change in the linguistic style used on @realDonaldTrump–one of the most important and influential social media accounts in history.

## Materials and methods

### Data

The corpus analysed for this study was sourced from the Trump Twitter Archive (http://www.trumptwitterarchive.com/) [32], which is a continuously updated archive of the tweets sent from the @realDonaldTrump Twitter account harvested directly from Twitter.com starting 2009-05-04. We downloaded the corpus from the archive on 2018-02-20, as well as a range of metadata, including the time stamp and the source (e.g. Web, Android, iPhone) for each tweet. Later, on 2018-07-27, we downloaded the retweet and favourite counts for each Tweet, which can change over time; these values do not factor into our main analysis. Because the aim of our study was to identify patterns of stylistic variation on the Trump Twitter account, we removed all retweets (11,303), leaving 21,739 tweets in our corpus, totalling 362,653 word tokens. This research project, including data collection, was approved by the University of Birmingham research ethics committee. The complete corpus is included in the Supporting Materials (see S1 File).

**Fig 1:**
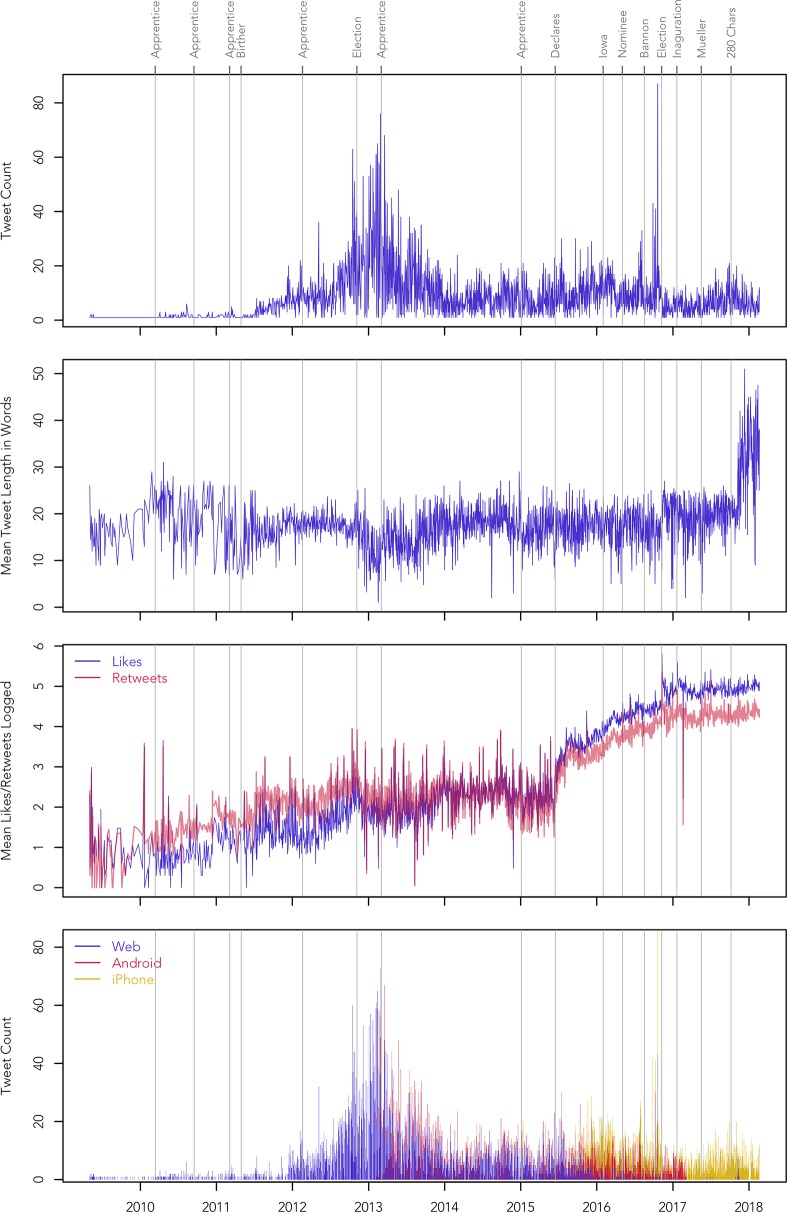
Change in activity on the @realDonaldTrump Twitter account.

Before describing the patterns of stylistic variation exhibited by this dataset, it is important to consider variation in activity on the account more generally to ground our linguistic analysis. The number of tweets sent from the Trump Twitter account per day varies considerably over time (see Fig 1). The account was used relatively infrequently for its first two years, but activity rose dramatically in the lead up to the 2012 elections, starting in March 2011, during the 11^th^ season of *All-Star Celebrity Apprentice*, when Trump briefly discussed the possibility of running for president and began to question Obama’s citizenship. The account was most active in February 2013: Trump was criticising president Obama, who had just begun his second term, as well as the talk show host Bill Maher, against whom he had filed a lawsuit, while also promoting the World Golf Championships, hosted at the Trump National Doral Miami, and the upcoming 13^th^ season of *All-Star Celebrity Apprentice*. After this point, activity on the account dropped off substantially, although Trump was still generally tweeting multiple times per day, a rate that would be roughly maintained for the rest of the period covered by the corpus, with smaller peaks following his declaration to run for the presidency in 2015, leading up to the Iowa Caucuses, and leading up to the general election.

The mean tweet length per day in the corpus is 17 words, but this rose to 33 words after Twitter increased the character limit for posts from 140 to 280 characters on 2017-11-07. Notably, we see no obvious change in the other metrics we observed around this date (see Fig 1) or in the results of our stylistic analysis of the Trump Twitter account, as described in the rest of this paper, although Twitter users more generally have been found to use more determiners and hashtags and fewer abbreviations immediately after this length increase [33]. There is considerable variability in the length of Trump’s tweets, with an interquartile range of 12 to 22 words before the Twitter length increase and 22 to 45 words after the length increase. Otherwise, there are no clear trends in tweet length over time (see Fig 1), although mean tweet length per day is moderately inversely correlated to the number of tweets sent per day (Pearson’s *r* = -.23): when a relatively large number of tweets are posted on the account on the same day, they tend to be shorter than when a relatively small number of tweets are posted.

The mean number of likes and retweets received by the Trump Twitter account per day rose substantially over time (see Fig 1, plotted using a logarithmic scale, base 10). Three main stages are apparent. First, both likes and retweets rose steadily from 2009 up to the 2012 election, when Trump was highly critical of Obama. During this period the mean number of retweets per day was generally larger than the mean number of likes. Second, between the 2012 election and Trump’s announcement in 2015 that he would run for the 2016 election, the mean number of likes and retweets per day remained relatively steady and comparable. Finally, after announcing his candidacy in 2015, the mean number of likes and retweets per day began to increase rapidly once again, with his tweets tending to receive more likes than retweets. This trend continues for the rest of the period covered by the corpus, although the rate of increase slowed after his inauguration.

Tweets posted on the @realDonaldTrump account originate from a range of sources, with the Twitter Web Client (11,252 tweets, accounting for 52% of the tweets in corpus), Android (4,659, 21%), and iPhone (4,224, 19%) being most common, together accounting for 93% of the tweets in the corpus. The remaining tweets in our corpus originate from 16 different sources, including TweetDeck (483), Instagram (133), Facebook (105), and iPad (48). Notably, the fact that social media management tools like TweetDeck account for such a small percentage of posts implies that Trump and his team usually tweeted in real time as opposed to scheduling posts ahead of time. There is also considerable variation over time in the use of the three main sources (see Fig 1): the Web Client was used almost exclusively up until 2013, after which both the Web Client and Android devices were used together until Trump declared his candidacy in 2015, at which point the use of the iPhone became increasingly common. By the time of the Iowa Caucuses at the start of 2016, the use of the Web Client had almost disappeared, with most posts originating from Android and iPhone devices. Finally, the use of the Android stopped entirely on 2017-03-25, not long after Trump’s inauguration, when Trump was advised to switch to an iPhone for security reasons [34].

**Fig 2:**
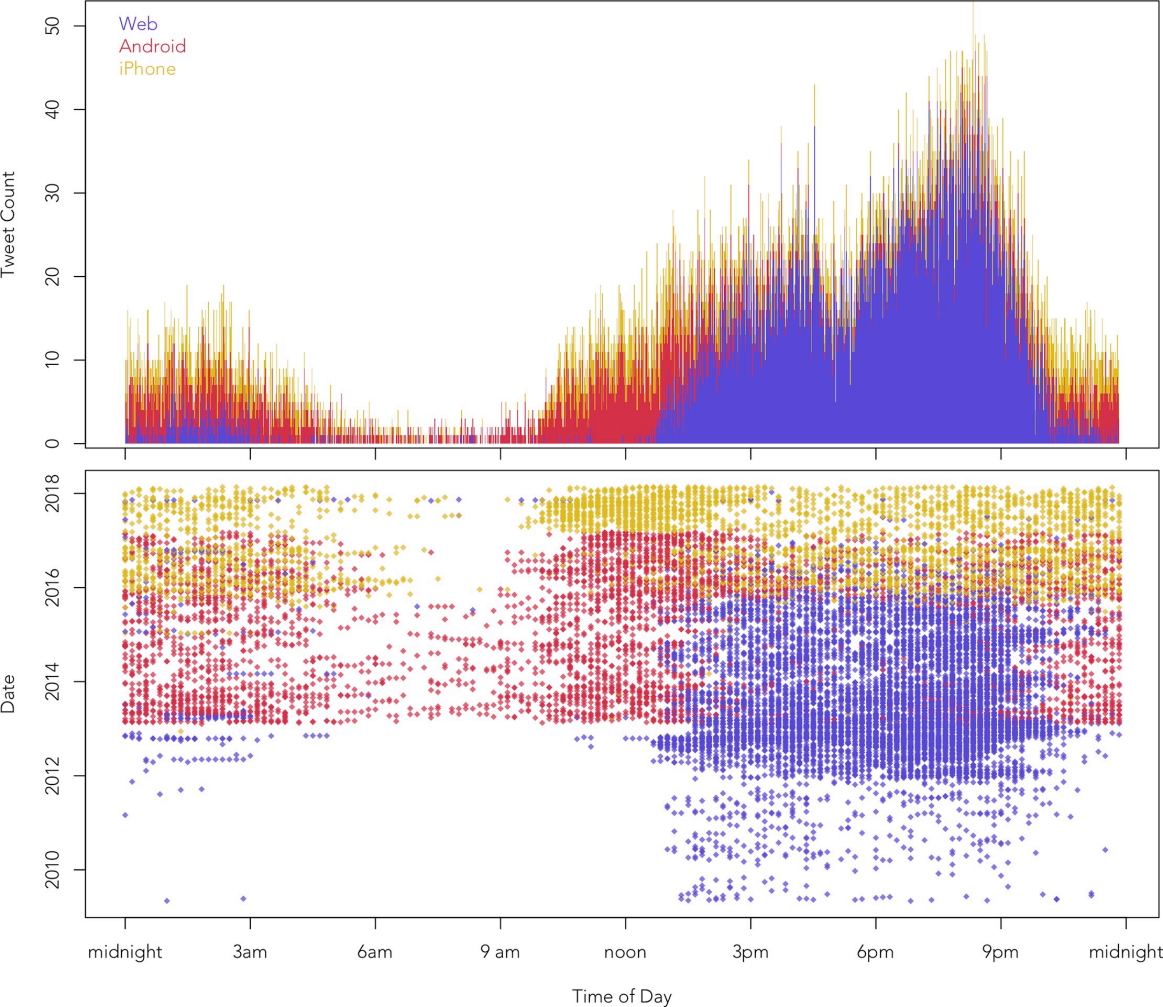
Change in device usage on the @realDonaldTrump Twitter account.

Activity on the account also varies considerably throughout the day, including in the use of particular devices (see Fig 2). In general, Trump tweets the most between around 12:00 and 22:00, with primary spikes in usage around 16:00 and 20:00. There is also a secondary spike in usage around 02:00. Alternatively, he is especially unlikely to tweet from about 05:00 to 10:00, when he presumably tends to be asleep. There is also generally a lull in tweets around 17:00, which may coincide with his evening meal. This pattern is especially true of tweets that originated from the web client and before 2016. Tweets from mobile devices tend to follow a similar pattern, although they are more likely to be used late at night and before noon, especially from Android devices, which were especially common during the campaign. It is important to acknowledge, however, that these timestamps do not always reflect Trump’s location at the time of tweeting. The timestamps returned by the Twitter API are provided in Greenwich Mean Time, which were then converted for the Trump Twitter Archive to Eastern Standard Time, which is the time zone where Trump is usually located. Our analysis is based on these adjusted time stamps; we did not attempt to make further adjustments, for example when Trump travelled, especially because many of his tweets are not associated with location information. Some inaccuracy is therefore unavoidable, but this has very little effect on our analysis, as we are ultimately focusing on much longer term trends on the Trump Twitter account.

### Measuring style

One way to describe stylistic variation in a corpus of texts is to focus on patterns of *linguistic co-occurrence*–seeing which linguistic features, especially grammatical features, tend to occur together in texts. For example, we know in general that English-language texts written in a more informal style tend to be characterised by relatively frequent use of contractions, interjections, and pronouns, whereas texts written in a more formal style tend to be characterised by relatively infrequent use of these features and relatively frequent use of nouns, prepositions, and determiners [35]. These differences exist because certain linguistic forms are more or less suitable for realising different communicative goals in different communicative contexts. For example, spontaneous modes of production (e.g. unplanned telephone conversations) generally encourage the use of more interactive features, while forms of carefully edited writing (e.g. academic articles) generally encourage the use of more informational features. Crucially, this conception of stylistic variation is amenable to statistical analysis, as style can be measured based on the use of linguistic features across the texts in a corpus. This general approach is common in register analysis [35], authorship analysis [36], and personality profiling [37].

*Multidimensional Analysis* (MDA) [35] is a standard method for the statistical analysis of stylistic variation in corpus linguistics [38]. Basically, MDA finds the main dimensions of stylistic variation in a corpus of texts by extracting common patterns of grammatical co-occurrence across the texts through a multivariate statistical analysis of the relative frequencies of a range of grammatical forms (e.g. parts-of-speech). Each dimension consists of a positive pole and a negative pole, where each pole is associated with a set of co-occurring grammatical features that tend to be in complementary distribution with each other–i.e. texts characterised by the frequent use of one set of features tends to be associated with the infrequent use of the other set of features. Crucially, this approach allows for multiple dimensions of stylistic variation to be identified in a corpus. For example, previous research has identified a series of dimensions of stylistic variation based on a factor analysis of the relative frequencies of a large set of grammatical features in a corpus representing a range of written and spoken genres [35]. One of these dimensions was interpreted as being associated with a narrative style, contrasting texts exhibiting frequent use of features such as past tense and third person pronouns, which are especially common in genres like fiction and biographies, with texts exhibiting frequent use of features such as present tense and attributive adjectives, which are especially common in non-narrative genres like news broadcasts and official documents. Other dimensions were associated with involved, persuasive, and abstract styles of communication. Similar approaches have been used to describe stylistic variation in other languages, including Nukulaelae Tuvulan [39], Korean [40], Somali [41], and Spanish [42]. MDA has also been used to describe stylistic variation within individual registers of the English language, including job interviews [43], call centre service encounters [44], and academic articles [45], as well as various forms of computer-mediated communication [46], including message boards [47] and blogs [48].

However, the analysis of Twitter data using MDA is problematic because tweets are so short, resulting in the relative frequencies of linguistic features in individual tweets being unreliable estimates of their relative frequencies in the population of tweets from which they are drawn. For example, in a tweet containing ten words, each individual word will have a relative frequency of at least once per ten words, but even the most frequent words in a larger Twitter corpus will come nowhere near this rate. Alternatively, every word that does not occur in that tweet will have a relative frequency of 0, even though many of those words would occur in a larger sample, often relatively frequently. Consequently, MDA is generally limited to texts over 500 words [49] or 1,000 words [50]–far longer than tweets, which in our corpus are on average 17 words long. Most previous MDA studies of short texts, including of Twitter [49, 51–53], have therefore combined individual texts to form longer texts suitable for frequency-based analyses. This approach is valid, if texts are combined in a principled manner, but it does not allow for variation to be examined at the level of the individual texts, drastically limiting the resolution and meaningfulness of these studies.

In this study, we analyse stylistic variation in the Trump Twitter corpus by employing a new form of MDA for short texts [54–57]. Rather than measure relative frequencies of linguistic features in each text, we simply record their presence or absence. Next, rather than subjecting this dataset to factor analysis, which is suitable for continuous data, we identify common patterns of variation using *Multiple Correspondence Analysis* (MCA) [58–59], which is suitable for categorical data. MCA is commonly used for analysing categorical survey data–to find groups of individuals who have answered questions similarly and to reveal the associations between the answers provided [60]. MCA has only been applied in a small number of studies in linguistics, for example to identify confounding variables in a corpus-based analysis of inflectional variation in Dutch [61], and to identify usage patterns in polysemic words [62], as well as in our own research on abusive language and trolling on social media [54, 56–57]. Outside our research, MCA has been used in very few studies of social media [63].

In this case, we use MCA much like factor analysis in traditional MDA–to reduce a large multivariate linguistic dataset down to a small number of the most important dimensions of variation that characterise that dataset in the aggregate. As in standard MDA, we then interpret these results stylistically based on the texts and the linguistic features most strongly associated with each dimension, as indicated by the coordinates and contributions returned by the MCA for each text and each linguistic feature for each dimension.

### Data analysis

To conduct a short-text MDA of the Trump Twitter Corpus, we first part-of-speech tagged each tweet in our corpus using the Gimpel Twitter Tagger, which is designed specifically for the grammatical analysis of Twitter [64]. For example, it identifies features that are specific to computer-mediated communication (e.g. hashtags, mentioning, URLs, emoticons), while also accounting for non-standard spelling and grammar. The Tagger only assigns 25 tags, which is too broad for a detailed MDA, so we enriched our feature set by searching the tagged corpus for occurrences of standard MDA features [35], such as pronoun types, verb tense and aspect, and passives. These features were originally selected based on a survey of previous research in usage-based linguistics [35], which identified a wide range of grammatical features in the English language whose relative frequencies in texts reflect their communicative function. In addition, following standard practice in MDA [48], we also searched for a variety of features related specifically to Twitter, including hashtags [65], initial and non-initial mentions [66], laughter acronyms [67], abbreviations [68], interjections [69], emoticons [70], emojis [71], capitalisation [70], punctuation [67], and pronoun and auxiliary omission [72]. In total, we identified 123 lexical and grammatical features. The tagged corpus (see S1 File) and the complete feature set (see S2 File) is included in the Supporting Materials.

To focus our analysis on the identification of robust patterns of stylistic variation, we removed all features that occurred in less than five percent of tweets [59], reducing our dataset to 63 linguistic features. We then subjected this 21,739-tweet-by-63-linguistic-feature categorical data matrix to an MCA to identify the main dimensions of stylistic variation in the Trump Twitter corpus. We also included tweet length, measured in words, as a supplementary quantitative variable in the MCA. Because we focused on the presence and absence of features as opposed to their relative frequencies, we did not directly control for text length, which is problematic because in general the more words a tweet contains the more likely it is to contain a variety of different linguistic features. Text length could have therefore confounded our analysis. Including tweet length as a supplementary variable allowed us to assess the degree to which variation across each dimension was predicted by text length, without affecting our main analysis. The dataset and R code we used to conduct the MCA is included in the Supporting Materials (see S3 File).

In the remainder of this paper, we discuss the first five dimensions returned by the MCA. Additional dimensions could be analysed in future research, but we chose to focus on these five dimensions because they are the strongest patterns identified by the analysis and because they are readily interpretable compared to later dimensions. In addition, these five dimensions account for 79% of the variance in our dataset, calculated using the standard adjustment for MCA [59], with a minor drop in variance explained after the fifth dimension. We also excluded the first dimension identified by the MCA from our main analysis of stylistic variation, because it primarily represents text length, as discussed in more detail below. The results of the MCA, including the contributions and coordinates for all features and all texts across the five dimensions, as well as the amount of variance explained by the complete model and the individual dimensions, are included in the Supporting Materials (see S3 File).

For each dimension, we interpreted the underlying stylistic pattern it represents based on two types of information. First, we considered the individual tweets most strongly associated with each pole of the dimension, as indicated by the coordinates and contributions for each text on that dimension. We refer to the positive and negative poles of each dimension, but it is important to stress that this is standard terminology and does not affect our interpretation of theses dimensions: these labels do not represent value judgements about the quality or semantic orientation of these dimensions; rather, the positive pole of the dimension represents the stronger of the two complementary co-occurrence patterns, although in general this difference is very small. In each case, we present two examples drawn from the five tweets that are most strongly associated with each pole of the dimension. In addition, the 100 tweets most strongly associated with each pole of each dimension are included in the Supporting Materials (see S4 File).

**Fig 3:**
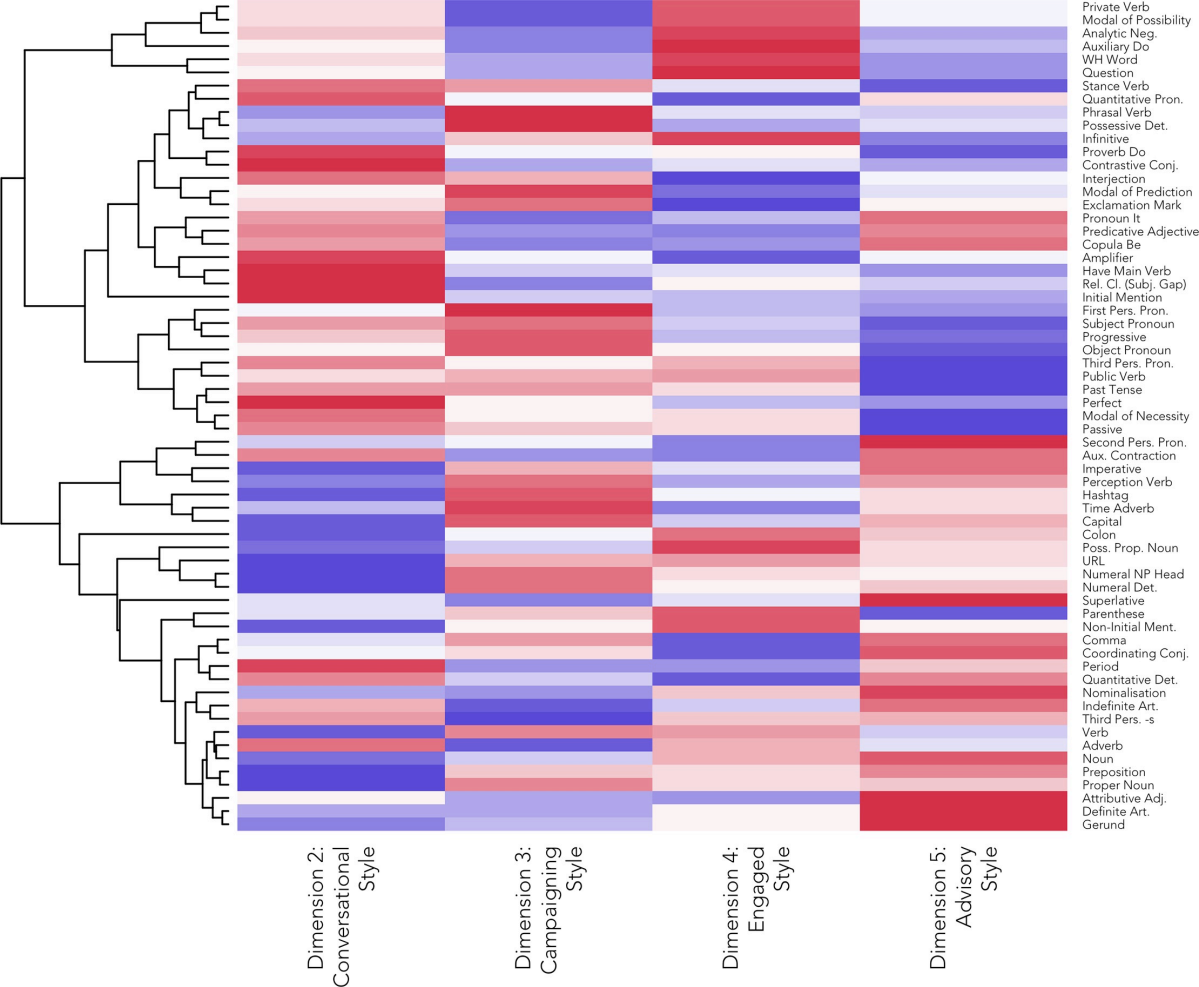
Linguistic feature coordinates across 4 stylistic dimensions.

Second, we considered the individual linguistic features most strongly associated with each pole of the dimension, based on the coordinates and contributions for each feature for that dimension. The heatmap presented in Fig 3 summarises the associations between the 63 linguistic features (rows) and each of the 4 stylistic dimensions (columns) based on the MCA coordinates. A red cell indicates that a feature is associated with the positive pole of a dimension, and a blue cell indicates that it is associated with the negative pole. The intensity of the cell colour represents how strongly that feature is associated with that dimension. For example, the linguistic features *initial mentions* and *have as a main verb* are strongly associated with the positive pole of Dimension 2, indicating that they tend to occur in Tweets with positive Dimension 2 coordinates, whereas *proper nouns* and *prepositions* are strongly associated with the negative pole of Dimension 2, indicating that they tend to occur in Tweets with negative Dimension 2 coordinates. The rows of this heatmap have been ordered using a hierarchical cluster analysis (complete-linkage clustering) based on the four sets of dimension scores, as reflected in the dendrogram reproduced on the left-hand side of the figure. This presentation does not affect our analysis. Our goal here is only to simplify the interpretation of the dimensions and to highlight linguistic features that tend to occur together across the 4 dimensions. For example, at the top of this heatmap, we can see that a number of verbal forms tend to pattern together, being relatively common in conversational and engaged tweets, but relatively uncommon in campaigning and advisory tweets.

**Fig 4:**
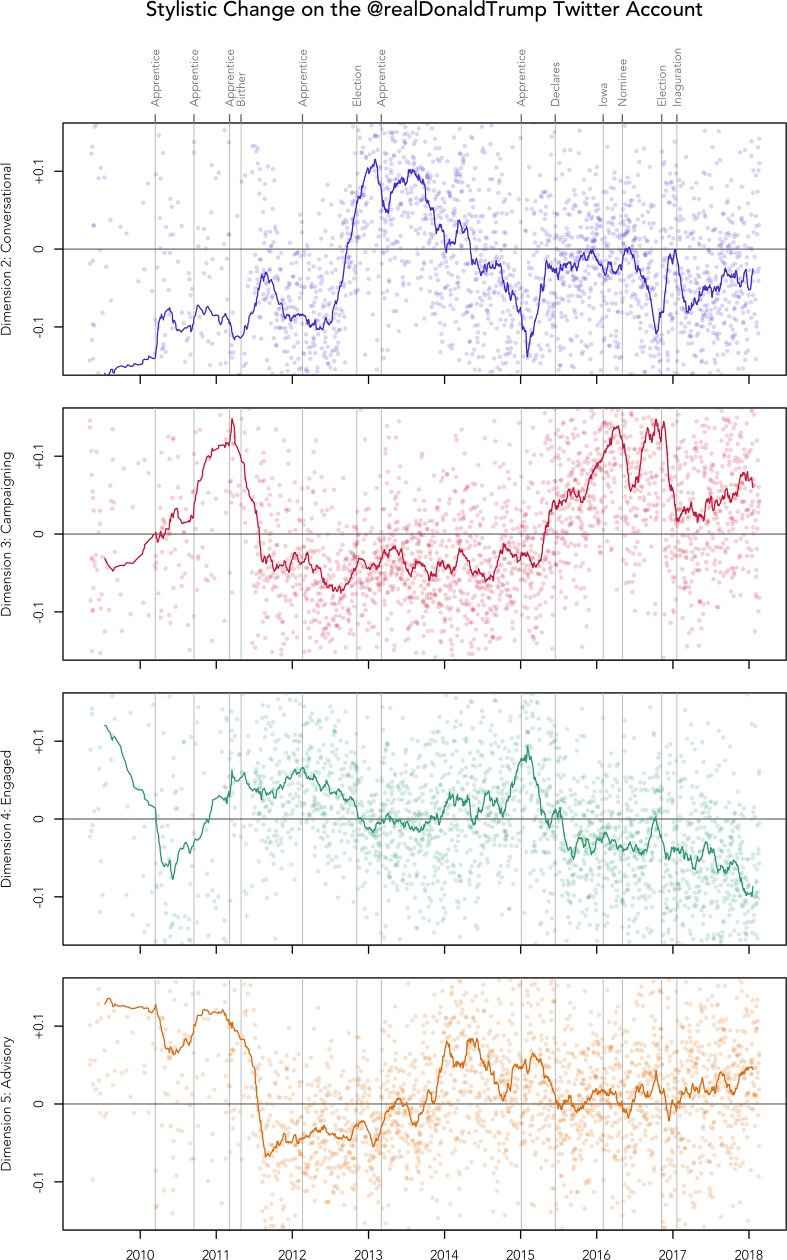
Stylistic change on the Trump Twitter account.

**Fig 5:**
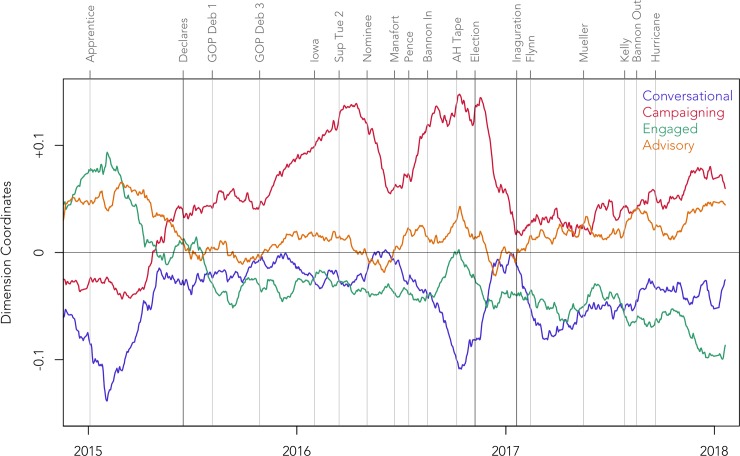
Stylistic change during the 2016 campaign on the Trump Twitter account.

We also tracked how the use of the four main dimensions of stylistic variation changed over time. To visualise patterns of stylistic change on the Trump Twitter account, we plotted the coordinates of each of the 21,739 tweets on each of the 4 main stylistic dimensions over the days in the corpus, as presented in Fig 4. To help visualise general trends in this dataset we also plotted 60-day moving averages by taking the mean coordinates of all tweets in that period on that dimension. We used a 60-day moving average to facilitate the identification of broad trends in the dataset; other windows gave similar results, although clear trends could have been obscured by taking very large windows. Notably, there are relatively few tweets per day before 2011, making trends during this period less meaningful. In addition, we plot the moving averages for the 4 dimensions together in Fig 5, zoomed in on the period around the campaign, to facilitate the analysis of stylistic shifts during this crucial period of time. Each time series is also annotated for important dates, so we can see what external events, if any, align with these major shifts in style. The R code we used to generate these figures is included in the Supporting Materials (see S3 File).

## Results

### Dimension 1: Tweet length

The first dimension returned by the MCA is primarily associated with variation in tweet length. For instance, example 3 is a tweet with a strong positive coordinate on Dimension 1 and is very long, whereas example 4 is a tweet with a strong negative coordinate on Dimension 1 and is very short:

3 ….Because of the Democrats not being interested in life and safety, DACA has now taken a big step backwards. The Dems will threaten “shutdown,” but what they are really doing is shutting down our military, at a time we need it most. Get smart, MAKE AMERICA GREAT AGAIN! (951790999784783872, 2018-01-12, D1: 0.865)
4 @DurangoRick Thank you:-) (352344945027330049, 2013-07-03, D1: -0.715)

**Fig 6:**
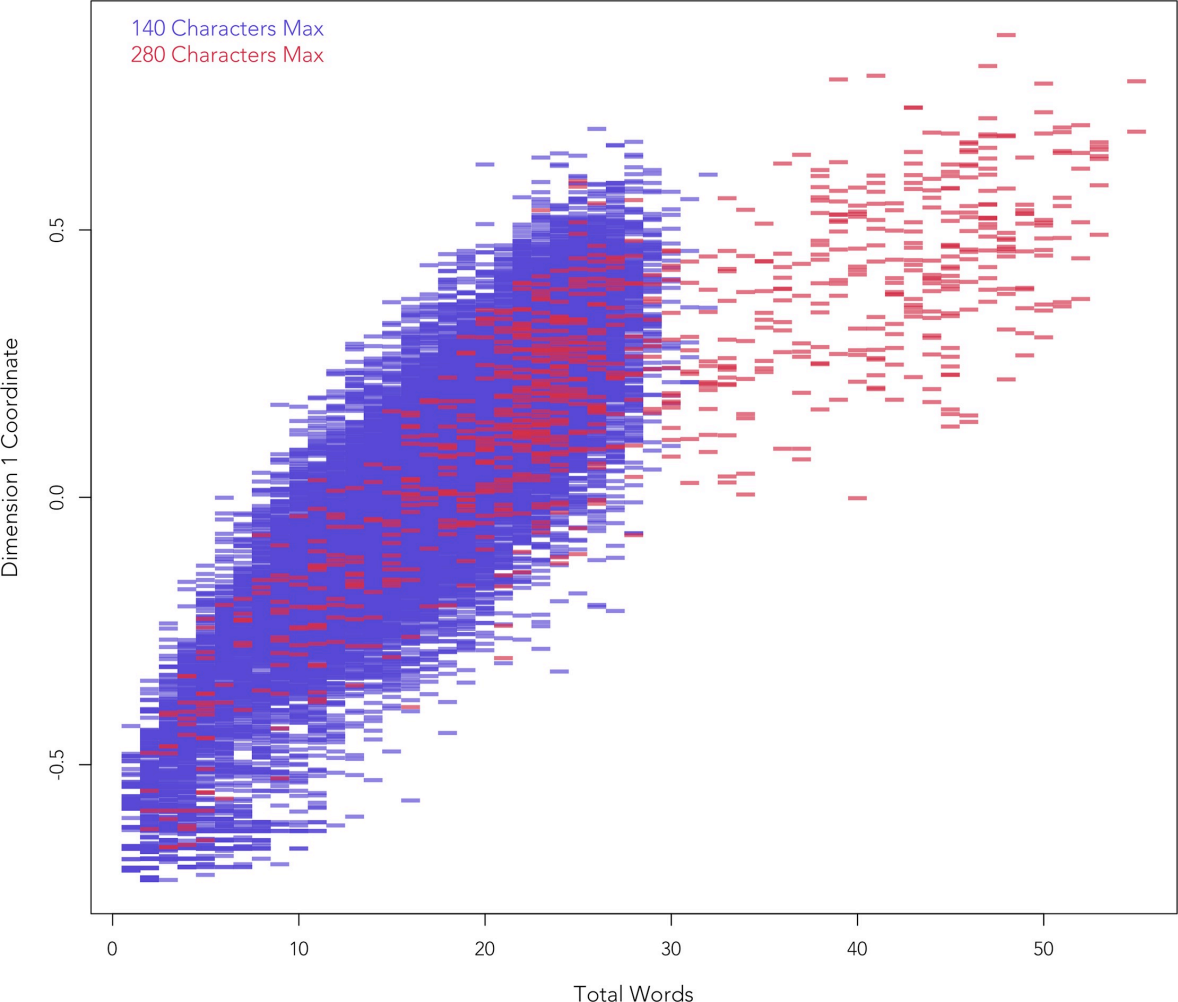
Dimension 1 tweet coordinate vs tweet length.

The correlation between text length measured in words and the Dimension 1 coordinates of the tweets is very strong (*r* = 0.87), with Dimension 1 coordinates clearly rising with text length, although the rise slows after tweets reach 30 words (see Fig 6), most of which were sent after the maximum length of the tweets was increased to 280 characters. This flattening occurs at least in part because as the length of tweets increases, the likelihood of new grammatical forms occurring for the first time decreases. This opposition between long and short tweets is also reflected in the linguistic features associated with this dimension, with the presence of 57 of the 63 features being associated with the positive pole of Dimension 1, indicating unsurprisingly that longer tweets tend to contain instances of more features. Only the presence of 6 features are associated with the negative pole of Dimension 1, including hashtags and URLs, which often stand alone in Trump’s shortest tweets.

While Dimension 1 is very strongly correlated with text length, the other four dimensions are only weakly correlated with text length (r < .15). The MCA has thus effectively isolated the effect of text length on Dimension 1, allowing us to largely control for variation in the length of tweets, despite the fact we did not directly factor tweet length into our analysis. We therefore exclude Dimension 1 from further stylistic analysis and focus on the interpretation of Dimensions 2 to 5 for the rest of this paper, confident that these dimensions are not confounded by variation in tweet length. We omit the time series for Dimension 1 from Figs 4 and 5 for this reason, but it is almost identical to the tweet length time series presented in Fig 1, which does not show an especially remarkable pattern, aside from a predictable increase following the raising of the maximum length of tweets in 2017.

### Dimension 2: Conversational style

Tweets with positive Dimension 2 coordinates tend to be interactive, often involving Trump directly conversing with other Twitter users. These tweets also tend to be informal, more similar in style to the spoken vernacular. For instance, examples 5 and 6 have very strong positive coordinates on Dimension 2 (0.653 and 0.629) and are both highly interactive and colloquial:

5 @RaydelMusic That's great—you will love what we are doing! (296718641175609344, 2013-01-30, D2: 0.653)
6 @SmuMom54 He should meet him and beat him—but that doesn't look like it's going to happen. (365841648430759938, 2013-08-09, D2: 0.629)

A number of the linguistic features most strongly associated with this pattern are present in these examples and are indicative of a conversational style. Notably, both of these tweets begin with *initial mentions*, which Trump often uses to converse with other Twitter users; this is the most highly contributing feature to this pole of the dimension. These tweets also tend to contain various types of *pronouns*, which are generally associated with interactive discourse [35], including *second person pronouns*, which are often used to interact with other accounts. Other Dimension 2 features associated with informal speech include *contractions*, *interjections*, *amplifiers*, *WH-words* and *question marks*, and *short sentences* (i.e. frequent use of full stops). Furthermore, these tweets tend to contain other features that have been found in previous MDA studies to be common in informal communication, including *auxiliary do*, *analytic negation*, and *predicative adjectives* [35].

Alternatively, tweets with negative Dimension 2 coordinates tend to be written in a more formal style, often intended to announce information rather than interact with users. For instance, examples 7 and 8 have very strong negative coordinates on Dimension 2 and are written in a more formal and expository style, at least for Twitter:

7 Congratulations to the 7 @TrumpCollection properties who made @USNewsTravel's Best Hotels List: http://t.co/1GgrePGJLM (560130576976723968, 2015-01-27, D2: -0.648)
8 Today in history WrestleMania 23: I shave @VinceMcMahon's hair—highest rated show in WWE history @WrestleFact http://t.co/7su88r1Mr0 (451080874201579520, 2014-04-02, D2: -0.588)

The features most strongly associated with these tweets are almost all linked by a more formal style–more informationally dense, composed of complex noun phrases, which generally require careful planning by the author [35]. These tweets not only tend to contain nouns of various types, including *proper nouns* and *nominalisations*, but also *noun pre-modifiers*, including a*ttributive adjectives*, *determiners*, and *numerals*, as well as *prepositions*, which are often used for *post-nominal modification*. Although these tweets tend to be far less interactive, they often contain *non-initial mentions*, which are generally used by Trump and his team to provide extra information about the people he is referring to, as opposed to interacting with them directly. Similarly, *hashtags* are used to enrich tweets with minimal expenditure of characters by linking tweets on the same topic together, while *URLs*, the most strongly associated feature with this pole of the dimension, essentially allow unlimited space to extend the content of the tweet.

Overall, Dimension 2 therefore represents an opposition between a more conversational style and a more literate style of tweeting–essentially a distinction between informal and formal tweets. Notably, this result reflects the two main forms of communication on Twitter: directed public conversation and the general broadcast of information to the entire network [15]. This basic pattern has also consistently been identified as the most important dimension of stylistic variation in MDA research on a wide range of languages and language varieties [73]. Replicating this result for Trump is therefore not especially surprising, but it does show that Trump varies his level of formality substantially. In addition, because our analysis aligns in this regard with previous MDAs, it provides a touchstone for our interpretations of subsequent dimensions.

To better understand this pattern of stylistic variation, we also considered how the use of this dimension changed over time (see Figs 4 and 5). From 2009 until 2012, the Trump Twitter account was relatively formal, aside from a spike in 2011 around the time of the Birther controversy. In these early tweets, Trump was often making announcements and consequently writing in a more informationally dense style, as illustrated in example 9:

9 Reminder: The Miss Universe competition will be LIVE from the Bahamas—Tonight @ 9pm (EST) on NBC: http://tinyurl.com/mrzad9 (3498743628, 2009-11-16, D2: -0.5)

Trump’s tweets became more informal in the lead up to the 2012 election, when he was a vocal critic of the administration, peaking around the start of Obama’s second term. In these tweets, Trump often interacted with users as illustrated in example 10:

10 @michellemalkin I fully supported McCain but when he lost, hoped Obama would be great for U.S.—he wasn't. (261526641493307394, 2012-10-25, D2: 0.51)

Use of this interactive style gradually decreased over the next few years, until a few months before Trump declared his candidacy, when there was a rapid rise in informality. His Tweets then remained relatively informal throughout the Republican primaries. However, after Trump secured the nomination, he reverted to a more formal style, as he shifted his focus to the general election. His more informal style returned in force only after the Access Hollywood tape was released, which risked derailing his campaign in its final month. Following the general election, we see another sharp rise in formality, although Trump grew more informal over the course of his first term.

Overall, variation over time on this dimension therefore appears to reflect variation in Trump’s intended audience: when he is promoting his brand to the general population or appealing to the general electorate he employs a more formal and expository style, but when he is speaking to his political base he employs a more informal and conversational style. Notably, this result is consistent with the sociolinguistic theory of *audience design*, which states that a speaker’s style, especially their level of formality, depends on the social background of their audience [74].

### Dimension 3: Campaigning style

Tweets with positive Dimension 3 coordinates tend to focus on promoting the Trump campaign–encouraging readers to vote for Trump, to join him and his supporters on campaign stops, and to access the campaign’s material online. For instance, examples 11 and 12 have very strong positive coordinates on Dimension 3 and are both clearly focused on promoting the campaign:

11 Thank you Las Vegas, Nevada- I love you! Departing for Greeley, Colorado now. Get out & VOTE! #ICYMI- watch here:… https://t.co/uIloPRtEn9 (792804645542305792, 2016-10-30, D3: 0.797)
12 Thank you Florida—we are going to MAKE AMERICA GREAT AGAIN! Join us: https://t.co/3KWOl2ibaW. #AmericaFirst https://t.co/vzKtRxzvwv (774435151975747584, 2016-09-09, D3: 0.754)

The features most strongly associated with these tweets include the use of *first person pronouns* in the *subject* and *object position*, as well as *possessive determiners*, marking these tweets as being highly self-oriented. These tweets also tend to contain *modals of prediction* and *time adverbs*, which are primarily used to promote and specify future plans, and *imperatives*, which are primarily used to encourage people to attend campaign events and vote. Moreover, these tweets often include *hashtags* to increase their visibility, which have been shown to be common in self-promotional tweets [75], as well as *capitalisation* and *exclamation marks*, which are used for emphasis.

Alternatively, tweets with negative Dimension 3 coordinates tend to be declarative and externally-focused, presenting Trump’s opinions and descriptions, both positive and negative, of people and events, as well as giving general business advice. For instance, examples 13 and 14 have very strong negative coordinates on Dimension 3 and are externally-focused, with no direct reference to Trump:

13 Obama's convention bounce is gone. @MittRomney has retaken the lead in the latest @RasmussenPoll http://t.co/yOw3U3ja (246315320871104512, 2012-09-13, D3: -0.508)
14 Mariano Rivera is greatest closer of all time. A leader in the club house and an exceptional man. One of the best @Yankees in history. (382213949069873152, 2013-09-23, D3: -0.478)

The features most strongly associated with these tweets include *third person singular verb forms*, which occur when the subject of the sentence is a person or thing external to the author. Similarly, these tweets often contain other features used to refer to external entities, including *possessive proper nouns* and *nominalisations*, as well as *predicative adjectives*, *copulas*, and *superlatives*, all of which are used to describe or assign characteristics to people or things [35].

Overall, Dimension 3 therefore represents an opposition between tweets that are intended to promote the campaign and tweets that have other communicative goals, including stating opinions on a wide range of topics. Unlike the Dimension 2, which is a pattern commonly found in MDA research, Dimension 3 identifies a very specific yet very important function of Trump’s Twitter account–as a communication outlet for his campaign.

This interpretation is clearly supported by change over time in the use of this dimension (see Figs 4 and 5). Most notably, Dimension 3 shows a clear shift towards a campaigning style right before Trump declared that he was running for president and has remained relatively high ever since. We also see internal fluctuations in the use of this style during this campaign. First, we see a strong gradual rise in the autumn of 2015, as Trump emerged as a front runner. Use of this style then fell slightly once Trump secured the Republican nomination before rebounding quickly after the Republican Convention, remaining very high until the election, at which point it dropped sharply once again, rising slowly only after the inauguration. Change over time in the use of this style of communication therefore appears to be closely related to the intensity of Trump’s presidential campaign.

It is also informative to look back through Trump’s timeline before the campaign began to see if we can perhaps find the roots of this campaigning style of discourse. Although this style was relatively uncommon for almost four years before Trump declared, it was used in some of his earlier tweets, when he was primarily using Twitter to promote his brand, as illustrated in examples 15 and 16.

15 He's hired! Listen to my #Apprentice Andy launch his radio show @AmericaNowRadio with me tomorrow 6PM ET http://t.co/xpz0oQa (100658594206322688, 2011-08-08, D3: 0.672)
16 Don't forget to watch me tonight on Late Night with Jimmy Fallon, 12:35 a.m. on NBC. I'll be making a big announcement! (25586441258012672, 2011-01-13, D3: 0.607)

Self-branding practices of this type have been found to occur frequently on social media not only by public figures but also by ordinary people as a way to achieve visibility and gain status [75]. This general form of discourse appears to be one with which Trump was familiar long before he ran for president and one that he used as a basis for his campaigning style.

### Dimension 4: Engaged style

Tweets with positive Dimension 4 coordinates tend to be engaged–not only interacting with other accounts, although that is often the case, but crucially acknowledging the ideas, viewpoints, and statements of others [76]. For instance, examples 17 and 18 have very strong positive coordinates on Dimension 4 and are directly engaging with other people and their perspectives:

17 You've got something unique to offer—find out what it is. Ask yourself: What can I provide that does not yet exist? Innovation can follow.. (301801582369071104, 2013-02-13, D4: 0.882)
18 I still don’t know who I’m going to choose. @GeraldoRivera or @LeezaGibbons? Who do you like? @ApprenticeNBC (566243577907662848, 2015-02-13, D4: 0.798)

The features most strongly associated with these tweets include *WH-words* and *question marks*, which are used in these tweets to ask questions–not only to interact with users and to seek their opinions, but as a way to reflexively recognise the viewpoint of others and suggest new ways of thinking. Similarly, *second person pronouns* are common in these tweets and are used both to interact with specific users and to reference their viewpoints. Additionally, these tweets often contain *auxiliary do*, *analytic negation*, *modals of possibility*, and *private verbs*, which are features that have previously been shown to be rhetorical resources commonly evoked to acknowledge alternative positions [76]. *URLs* are also used frequently in these tweets, usually to engage directly with external web content.

Alternatively, tweets with negative Dimension 4 coordinates tend to provide categorical judgements about other people, events, institutions, and policies, without marking the claims as subjective opinions, much less engaging with the opinions of others. Essentially, there is no acknowledgment in these tweets that the statements being made are subject to debate [77]. For instance, examples 19 and 20 have very strong negative coordinates on Dimension 4 and both make unqualified claims:

19 I will be campaigning in Indiana all day. Things are looking great, and the support of Bobby Knight has been so amazing. Today will be fun! (727142563010891776, 2016-05-02, D4: -0.462)
20 All signs are that business is looking really good for next year, only to be helped further by our Tax Cut Bill. Will be a great year for Companies and JOBS! Stock Market is poised for another year of SUCCESS! (945780569388015616, 2016-12-26, D4: -0.456)

In both examples Trump declares something to be unambiguously true–positive evaluations of his campaign and the stock market–even though these claims were not generally accepted as true at the time. These two tweets also contain many of the most indicative features of this unengaged style: *attributive* and *predicative adjectives* and the *copula* tend to be used by Trump to describe subjects in absolute terms with maximum authorial investment, without entertaining the possibility of alternative descriptions or viewpoints, while *amplifiers*, *superlatives*, *interjections*, *capitals*, and *exclamation marks* tend to be used to emphasise the certainty of the point being made, contributing to a hyperbolic and uncompromising style. It is also notable that example 19 exhibits a campaigning style (D3: 0.103). This result demonstrates one of the advantages of a multidimensional approach to the analysis of stylistic variation: individual texts can be scored highly on multiple dimensions, reflecting the fact that texts, even as short as tweets, can accomplish numerous communicative goals at the same time.

Overall, Dimension 4 therefore represents an opposition between an engaged or *heteroglossic* style, where Trump is acknowledging the viewpoints of others, and a disengaged or *monoglossic* style, where he is expressing his opinions as if they are statements of fact. Like Dimension 3, this dimension has not been identified in previous MDA research, although there is a clear basis for its interpretation in linguistic appraisal theory [76]. Its prominence in this corpus suggests that it is an especially important stylistic pattern for Trump.

Dimension 4 shows a relatively simple trend over time (see Figs 4 and 5), with a gradual fall in engagement since 2011, aside from spikes around the time of various seasons of *The Apprentice*, especially the final season in 2015, not long before Trump would declare. His degree of engagement shows an especially strong fall immediately after he declared, reflecting a steady stream of tweets categorically asserting the inevitable success of his campaign. Engagement did, however, rise briefly after Steve Bannon joined the campaign, as illustrated in example 21:

21 The failing @nytimes reporters don't even call us anymore, they just write whatever they want to write, making up sources along the way! (787425145489072128, 2016-10-15, D4: 0.535)

But this somewhat more engaged style did not last long. After the Access Hollywood tape was released, Trump increasingly adopted a more disengaged style, as in example 22, which was singled out by the media as being “almost-wilfully ignorant” [78]:

22 The attack on Mosul is turning out to be a total disaster. We gave them months of notice. U.S. is looking so dumb. VOTE TRUMP and WIN AGAIN! (790337063489040384, 2016-10-23, D4: -0.307)

Furthermore, Trump’s tweets became even more disengaged since he was elected, with engagement reaching its lowest levels at the end of the period covered by this study. Notably, this finding echoes what has been described as ‘the power paradox’–how the skills important for obtaining power, including the ability to consider other people’s points of view, often deteriorate once power is secured [79].

### Dimension 5: Advisory style

Tweets with positive Dimension 5 coordinates tend to involve Trump providing advice to his followers–most often offering general guidance on life and business as illustrated by example 23, although sometimes this advisory tone is used to convey more personal messages as illustrated by example 24:

23 Have your own vision and stick with it. Don't be afraid to be unique. Every day is an opportunity to show what you can do at the highest level. (420309480987836416, 2014-01-06, D5: 0.757)
24 Sorry losers and haters, but my I.Q. is one of the highest -and you all know it! Please don't feel so stupid or insecure,it's not your fault (332308211321425920, 2013-05-09, D5: 0.745)

The features most strongly associated with these tweets include *imperatives*, which are generally used in a consultative and motivational manner. These tweets also tend to contain frequent use of pronouns, including the *third person pronoun it*, which is used to make general claims, and the *second person pronoun you*, which is used to directly reference the audience being advised, often in a generic way. In addition, these tweets often contain features such as *predicative adjectives*, *copula be*, *superlatives*, *private verbs*, *perception verbs*, and *modals of possibility*, all of which tend to be used to qualify the advice being provided.

Alternatively, Tweets with negative Dimension 5 coordinates tend to do the exact opposite, often involving Trump commenting critically on the actions or decisions of particular people. For instance, examples 25 and 26 have very strong negative coordinates on Dimension 5 and are highly critical of others:

25 Who handed Iraq over to Iran yesterday? @BarackObama. We have gotten nothing from the Iraqis—we should have them pay us back with oil. (146684909610741760, 2011-12-13, D5: -0.580)
26 Why did @DanaPerino beg me for a tweet (endorsement) when her book was launched? (612063082186174464, 2015-06-20, D5: -0.576).

The features most strongly associated with these tweets include various markers of impersonal narrative. *Third person pronouns* are the most indicative feature of this style and are generally used to refer to other people, who may be referenced multiple times in the text, including through *mentions*. More generally, *pronouns* are common both in *subject* and *object* position. Other narrative features are also common, including *past tense*, *perfect aspect*, and *public verbs*, which are used to report on completed actions, and *progressive verbs*, which are used to refer to events in progress [35]. These tweets, however, are not simply mini-narratives: they often describe events, but crucially they tend to provide a critical assessment of the actions of the actors involved. Critiques are realised using a range of rhetorical strategies: *self-reference* to introduce Trump’s alternative, especially in a more positive light than his targets, signalling an “us versus them” dichotomy [80]; *modals of necessity* to mark the previous actions under attack; *passive constructions* to emphasise the patient of the act [80]; and *rhetorical questions* to set up attacks and place blame.

Overall, Dimension 5 therefore represents an opposition between a more advisory and a more critical style of communication. Both styles are essentially interactive and outward looking: one is giving general advice to his audience, whereas the other is critiquing individual people for past and current actions.

Change over time in this dimension offers further support for this basic interpretation (see Figs 4 and 5). Initially, the account was highly advisory, often framing Trump as an expert in giving business advice, but there was a clear increase in critical tweets from 2011 to 2012, from the start of the Birther controversy and throughout Barack Obama’s campaign for his second term, as illustrated in example 27:

27 @BarackObama sold guns to the Mexican drug cartels. They were used in the murders of Americans. Where is the outrage? (121618605820481536, 2011-10-05, D5: -0.492)

Notably, this pattern aligns with Trump’s books, which at first primarily gave business advice, but grew to be more political and critical over time. The degree of criticalness remained very high until after the 2012 election, at which point it gradually fell once again, never returning to these levels. However, not long before Trump declared his candidacy, his use of this critical style rose back up to middling levels. It remained relatively stable ever since, although it fell briefly when Bannon took over, only to return after the Access Hollywood tape was released, mirroring Dimension 4.

## Discussion

Based on a quantitative and qualitative analysis of grammatical co-occurrence patterns, we identified four main dimensions of stylistic variation in the tweets sent from the Donald Trump Twitter account between 2009 and 2018. We interpreted these four dimensions as representing variation in conversational, campaigning, engaged, and advisory styles of discourse. We also tracked how the tweets in the corpus varied over time across these four dimensions, to see how the style of the account changed. All four dimensions showed clear temporal patterns and most major shifts in style align to a small number of indisputably important points in the Trump timeline, especially the 2011 Birther controversy, the 2012 election, his 2015 declaration, his 2016 Republican nomination, the 2016 election, and his 2017 inauguration, as well as the seasons of his television series *The Apprentice*.

Given these results, we believe our analysis not only provides a meaningful and holistic description of stylistic variation and change on the Trump Twitter account, but also offers evidence that there was a communication strategy underlying the use of this platform by Trump and his team, especially during the 2016 campaign. We see clear shifts in the way the campaign uses Twitter depending on their general communicative goals, including appealing to different audiences, promoting the campaign, defending Trump against criticisms, deflecting controversies, and attacking opponents–all of which are fundamental to successful political campaigns [81]. The claims commonly found in the media that Trump’s Twitter posts were lacking in strategy [82–83] are difficult to reconcile with these results. That is not to say we believe that Trump or his team fine-tuned each tweet character-by-character to exhibit the intricate grammatical patterns identified in our analysis. The amount of care that was given to composing each tweet is probably unknowable. But we do believe these stylistic patterns reflect decisions made by Trump and his team about how to run their campaign and how to use social media as a communication platform. In particular, based on our stylistic analysis of the language of the Trump Twitter account, we propose four hypotheses about how Trump and his team were able to use Twitter effectively during the candidacy.

First, Trump’s Twitter communication style appears to have shifted depending on his intended audience, specifically becoming more informal and conversational when he was trying to appeal to the Republican base and members of the public who shared his political views, and becoming more formal and informationally dense when he was trying to appeal to the general public. Most notably, his tweets were substantially more conversational during the Republican primaries compared to the general election. This strategy was perhaps especially useful for attracting working-class voters, who are generally thought to have been largely responsible for Trump’s victory [84], although this view has been disputed [85]. These voters may have preferred Trump’s informal, unguarded, and outspoken style compared to his competitors in the Republican primaries. Alternatively, shifting to a more formal style during the general election may have helped Trump attract enough independents and moderates to secure his narrow victory over Clinton.

Second, Trump and his team appear to have employed a deliberate and sustained campaigning style on Twitter throughout the election period. Although it was not the only form of discourse employed on the account during the campaign, this direct and unapologetic form of self-promotion dominated. Notably, this style appears to have been based on Trump’s earlier attempts at promoting himself as a celebrity and businessman on social media. This experience may have given him an advantage in the 2016 election. Whereas career politicians may have had trouble promoting themselves effectively on social media, Trump’s experience may have helped him to stand out among more traditional politicians, and portray a confident, authentic, and distinct online persona. Crucially, this unique promotional style of communication may have also helped Trump attract new voters who were more familiar and comfortable with this social media style of language than traditional forms of political discourse.

Third, Trump and his team appear to have countered critical coverage of the campaign by disengaging from other viewpoints, focusing instead on using social media to express opinions, attack opponents, and promote the campaign. Rather than debating his critics or disputing their claims, Trump often ignored their attacks and continued to present his own agenda, doubling down on controversial views, especially as the campaign progressed. This defensive strategy may have insulated Trump and his supporters from these attacks, while helping to present Trump as an outsider who was standing up to mainstream politicians and media outlets. For example, after the Access Hollywood tape was released, which was probably the low point for the campaign, there was a sharp drop in engaged discourse on the account, as Trump and his campaign withdrew from debate, and notably increased their attacks on the Clintons. This decision may have been a crucial moment in the campaign: by minimising his engagement with this controversy and generating new controversies for his opponents, Trump was perhaps able to keep his base of support from disintegrating.

Fourth, Trump and his team appear to have struck a balance between using Twitter for criticism and promotion. During the campaign, Trump regularly posted tweets written in a highly critical style, directly and often harshly attacking people for their past and current actions–not only his political opponents, but a wide range of other targets. Critical tweets certainly appear to have been an important part of the campaign’s communication strategy. However, despite media reports highlighting this aspect of the campaign [27], critical tweets did not dominate his timeline, which generally focused on promoting his campaign and expressing his own political positions. This hypothesis is supported by previous research on sentiment analysis, which found that Trump ran a more positive campaign than Clinton on Twitter in the ten days before the election, using more positive words and generating a greater positive sentiment around the campaign [15]. This strategy may have also further undermined negative coverage of Trump in the media, which often focused on Trump’s more critical tweets, implying incorrectly that this was the only form of communication used on the account. Trump and his team appear to have struck an important balance, which may have helped them navigate their way to victory in the general election.

Finally, in addition to these four specific hypotheses about how Trump and his team used Twitter effectively during the campaign, we also believe our analysis may allow us to identify when Trump decided to run for the presidency. We know that Trump declared on 2015-06-16 and that he had previously formed an exploratory committee on 2015-03-17, but we do not know exactly when he personally decided that he would run for president. Our basic argument in this paper is that changes in Trump’s communication style over time reflect changes in his communicative goals, and more specifically that shifts during the campaign reflect shifts in the political goals of Trump and his team. We therefore might expect to find shifts in the style of language used on the account around the time Trump decided to run.

In fact, we see one especially clear yet unexplained inflection point across multiple dimensions around 2015-02-03, not long before Trump formed his exploratory committee. As the final season of the *Apprentice* was drawing to a close, Trump’s tweets became much more conversational and much less engaged. Trump also directly referenced running for President on this day:

28 Let’s together Make America Great Again! Vote Trump at https://t.co/YoNf60s0lm (562414915625832000, 2015-02-03)

The URL in this tweet links to an unofficial poll posted on the website poll.fm asking people for ‘your vote for Republican presidential candidate’, for which Trump received 5% of the 449,964 votes. Remarkably, of the 161 tweets sent from the account in February 2015, as well as the 302 retweets sent over this period, this is the only tweet sent from an iPhone. Given that Trump used an Android during this period, this tweet may have been sent on Trump’s behalf by someone on his soon-to-be campaign team. Furthermore, out of the 462 occurrences of ‘Make America Great Again’ in the complete Trump Twitter timeline, this is only the 23rd occurrence. There is also a notable retweet on this day, which is no longer available:

29 “@PharmStudents18: Next president of the United States of America. @realDonaldTrump #Trump2016 you've got my vote.” (562417684055617000, 2015-02-03)

Although clearly far from conclusive evidence that Trump decided to run at this time, these tweets, along with the sharp changes in style we observe, are suggestive of a shift in Trump’s political outlook.

We also see a second inflection point across multiple dimensions around 2015-04-07, not long after Trump formed his exploratory committee, with Trump’s tweets starting to become substantially more promotional and critical. Ultimately, these shifts foreshadowed Trump’s general style of communication during the campaign, at least through the Republican primaries. Once again, his Tweets from around this time are suggestive. For example, on this day, Trump hinted once again that he might run:

30 Wow the respected Monmouth University poll has me ahead of most Republican candidates nationwide and most people don't think I'm running! (585406176541728000, 2015-04-07)

Throughout this week he also retweeted dozens of posts from others encouraging him to run. Perhaps even more notably, however, Trump was in the news this very day for travelling to Iowa–the first state to vote in the Republican race. While there, he not only gave a press conference from his plane in Des Moines and delivered a speech on education at a performing arts centre in Indianola, but he hired three Iowa-based political operatives. In hindsight, it seems Trump had decided to run by this date at the latest, and that this decision was echoed by a shift in his tone on Twitter, especially by a rise in self-promotional discourse.

## Conclusion

In many ways, Twitter was Donald Trump’s primary communication platform during the 2016 Republican primaries and presidential campaign. He used Twitter to communicate with his supporters, the electorate, the media, and the world on a daily basis, with news cycles often being driven by a selection of the most controversial tweets from his timeline. Given his election victory, Trump’s Twitter account demands analysis, as it provides one example of how to run a successful social media campaign. Regardless of one’s political persuasion or one’s opinion of Donald Trump, we believe it is of critical importance to understand the unique and ultimately effective communication strategy Trump and his team implemented on social media during the 2016 campaign.

In this paper, we have argued that one way to understand Trump’s communication strategy is through the careful multivariate linguistic analysis of his tweets. We found that the style of his tweets varied systematically over time depending on the communicative goals of Trump and his team. On the whole, our results point not only to the value of running a balanced social media campaign–simultaneously promoting one’s candidacy, communicating one’s platform, opposing one’s critics, and attacking one’s opponents–but of constructing a confident and distinctive online persona. Understanding this social media strategy is an important part of understanding the Trump campaign–and more generally of understanding how to successfully run or for that matter how to successfully oppose political campaigns online, which are becoming a crucial part of modern politics.

Although the focus of this paper has been on the 2016 campaign, it is also important to understand how the Trump Twitter account has been used as a communication platform by the administration. In fact, one of the clearest shifts in style in the entire corpus occurred once Trump won the presidency: at that point his style across all four dimensions shifted dramatically. Over the first year of the Trump administration, which includes the start of the Mueller investigation and the Charlottesville tragedy, we see a more consistent style of communication; these events appear to have had relatively little effect on the style of the account compared to major events during the campaign. In general, we simply see a gradual fall in an engaged style and a gradual rise in campaigning style after Trump assumed office, perhaps reflecting decreasing concern from Trump and his team about how they are perceived by people from outside their base or an increasing concern about running for a second term. A more focused analysis of stylistic variation and change on the Trump Twitter account during his presidency would allow for more detailed patterns to be identified that could help us better understand the strategy of the administration, much as we have been able to better understand the strategy of the campaign.

Finally, this study has demonstrated how a detailed and objective linguistic analysis can be used to better understand important societal issues. With the rise of the internet and social media, world events are being driven by online communication, which generates huge amounts of text that must be analysed to understand these events and the forces that drive them. For example, in terms of the Trump presidency, the Democratic National Committee hack, the *New York Times* anonymous op-ed, and the fake news crisis are all major news items that are directly linked to collections of online language data. This new communicative landscape has led to a proliferation of what are essentially linguistic analyses being published in the popular press, hoping to make sense of internet chatter. These analyses, however, have largely been conducted by journalists and data scientists; from a linguistic perspective they are superficial at best and often appear to be politically biased. This need not be the case. The theories and methods of linguistics–including corpus linguistics, forensic linguistics, sociolinguistics, and discourse analysis–offer a foundation for linguists to make sense of these large collections of textual data, rather than to simply increase the amount of noise online. This is a challenge linguists must accept.

## Supporting information

S1 File Data. This file contains the Twitter corpus used for this study in raw and tagged form.
(ZIP)

S2 File Features. This file contains information on the set of linguistic features analysed in this study.
(ZIP)

S3 File Analysis. This file contains the full dataset, R code, and output for the quantitative analysis conducted for this study.
(ZIP)

S4 File Examples. This file contains the 100 tweets with the highest and lowest coordinates on each of the five dimension, as well as coordinates for the full set of tweets in the corpus scored on the five dimensions.
(ZIP)
