# Supplementary material for: Stylistic variation on the Donald Trump Twitter account: A linguistic analysis of tweets posted between 2009 and 2018
Source: PLoS One. 2019 Sep 25;14(9):e0222062. doi: 10.1371/journal.pone.0222062 (PMC6760825; doi:10.1371/journal.pone.0222062)
Supplement: S2 File — This file contains information on the set of linguistic features analysed in this study. (ZIP) [file pone.0222062.s002.zip › FEATURE_SET/Full_Feature_Set_TRUMP.pdf]

Feature_Set	Tag	Feature	Description	Example
Acronym	ACRONYM	Acronym	Refers to any initials separated by full stops	U.S.A, U.S, N.Y.
Quantifier-Adverb	ADVQUAN	Quantifier as adverb	Refers to quantifiers which are functioning as adverbs	You all are my inspiration, We are all happy to see you
Amplifier	AMPLIFIER	Amplifier	Refers to adverbs used to intensify the verb/adjective	very, absolutely, so
Analytic-Neg	ANALNEG	Analytic negation	Refers to 'not' plus contracted forms	can't, cannot, not
Attributive-Adjective	ATTRIBADJ	Attributive Adjective	Adjectives that come before the noun and any other adjective not tagged as predicative	The *big* cat
Auxiliary-Do	AUXDO	Auxiliary do	Refers to any form of DO that is followed by (up to three adverbs and) a verb	I *do* not like cheese, I *did* take the bins out
BE-initial-verb	BEIVB	BE as initial verb	Refers to when BE is the first verb in the Tweet (after initial mentioning) or if BE is the first verb after a full stop	
Copula BE	BEMV	BE as a main verb	Refers to when BE is the main verb and when BE is in its copular form; that is, when it is followed by a predicative adjective	She *is* a beautiful woman; She *is* beautiful
Bracket	BRACKET	Brackets	Refers to the use of brackets	
Capital	CAPS	Capitalisation	Refers to two or more capital letters that is not tagged as an acronym/URL/mentioned username	
Cause Subordinator	CAUSESUB	Cause subordinators	Refers to subordinators which indicate a causal relationship	because
Coordinating conj.	CCONJ	Coordinating conjuncts	Refers to coordinating conjunctions	and, &
Contrastive Conj.	CNTRSTCONJ	Contrastive conjuncts	Refers to conjunctions that signal a contrast is being made	but, by contrast
Colon	COLON	Colon	Refers to the use of colons	:
Comma	COMMA	Comma	Refers to the use of commas	,
Comparative	COMPARATIVE	Comparatives	Refers to adjectives in their comparative	She is *better* today, Drake >

			form	RiRi, I went for something *more* substantial
Concessive Subordinator	CONCESUB	Concessive subordinators	Refers to subordinators which indicate concession	although, though
Conditional Subordinator	CONDISUB	Conditional subordinators	Refers to subordinators indicating a condition	if, unless
WH-contracted verb	CONTRACTWH	WH words contracted	Refers to WH that have the verb contracted	*what's, who'd, where's*
Other Copula	COPVB	Copula verbs	Refers to copula verbs but not BE as a main verb (even if it is in its copula form, that is: when it is followed by predicative	
Definite Article	DEFART	Definite article	Refers to the use of the definite article	
Demonstrative Determiner	DEMDET	Demonstrative determiner	Refers to the use of this, that, these, those as a determiner; that is followed by noun	
Demonstrative Pronoun	DEMPRO	Demonstrative pronoun	Refers to the use of this, that, these, those as a pronoun; that is NOT followed by noun	
Quantifier-Determiner	DETQUAN	Quantifier as determiner	Refers to quantifiers used as a determiner	*Few* people, *some* people
DO-initial-verb	DOIVB	DO as initial verb	Refers to when any form of DO is the first verb in the Tweet (after initial mentioning) or if DO is the first verb after a full stop	Doesn't the world look different
Downtoner	DOWNTONER	Downtoner	Refers to adverbs used to reduce the force of the adjective/verb	He is *slightly* fat, It was *pretty* awful
Elipsis	ELIPS	Ellipsis	Refers to three or more fullstops	…
Emoji/Emoticon	EMOTICON	Emoticons or emojis	Refers to anything tagged by the Gimpel tagger as an emoticons	:)
Exclamation Mark	EXCLAM	Exclamation marks	Refers to the use of exclamation marks	!!!, !?
Existential there	EXIST	Existential *there*	Refers to the use of *there* in its existential form and thus not as a place adverb	*There* was a man in dark clothing, *There* may be 5 or 6 obstacles
First Pers. Pron.	FSTPP	First person pronoun	Refers to pronouns:	I, We, us, me, myself,

			subject/object/possessive/reflexive and possessive determiners that refer to the first person: singular and plural plus contracted forms	ourselves, ours, our, my, mine
Period	FULSTOP	Full stop	Refers to the use of full stop	.
Gerund	GERUND	Gerund	Refers to prepositional complement: when a preposition is followed by noun in -ing form (but this is tagged by Gimpel tagger as a verb)	Sarah talked about *leaving* her job
BE going to construction	GOINTOBE	The form 'BE going to'	Refers to any form of BE (including contracted) + going + to	I*'m going to be* in Kansas tonight, She *is going to* leave her job.
Hashtag	HASHTAG	Hashtag	Refers to the use of Hashtag	
HAVE-initial-verb	HAVEIVB	HAVE as initial verb	Refers to when any form of HAVE is the first verb in the Tweet (after initial mentioning) or if HAVE is the first verb after a full stop	
Have Main Verb	HAVEMV	HAVE as main verb	Refers to when any form of HAVE is the main verb	She *has* so much money, I *had* seven chocolates
Indefinite Article	INDEFART	Indefinite article	Refers to use of indefinite article	a, an
Imperative	IMPERATIVE	Imperative clause	Refers to clauses in imperative mood	Go away!, Don't be foolish!
Infinitive	INFINITIVE	Infinitive	Refers to verbs in infintive form that is not adjective + to complement clause or split infinitive	to be, to have
Verb-ING	ING	Verb in ING form	Refers to verb in ING form that is not in standard progressive form (likely a gerund/nominalisation)	Going for walks is my favourite thing to do on a Saturday
Pronoun IT	IT	Pronoun IT	Refers to any form of pronoun IT: contracted, reflexive, possessive and possessive determiner	It is great, it's okay, itself, its
Initial verb	IVB	Initial verb	Refers to initial verbs in their base form which are followed by particular things making them unlikely to be imperative	wish you were here, love to go, want to spend, do you

			clauses	
Laughter	LAUGH	Laughter	Refers to written out laughter	haha, lol, lmao, lmfao
Modal of Necessity	MDNEC	Modal of necessity	Refers to modals indicating necessity/obligation	should, mustn't, ought
Modal of Possibility	MDPOSS	Modal of possibility	Refers to modals indicating probability/possibility/ability	can, may, mightn't
Modal of Prediction	MDPRED	Modal of Prediction	Refers to modals indicating prediction	will, shall, I'll
Non-initial Ment.	MENTION	Non-initial mentioning	Refers to mentioning that is not initial	\@username
Phrasal verb	MULTIWVB	Multiword verb	Refers to both prepositional and particle verbs	
Negative interjection	NEGINTJ	Negative interjections	Refers to forms of NO that are tagged as interjections by Gimpel tagger	No_!, Naaaa_!
Nominalisation	NOMIN	Nominalisation	Refers to when verbs/adjectives are converted into nouns	action, statement
Numeral Determiner	NUMDET	Numeral as determiner	Refers to use of numerals functioning as determiners	Three dogs
Numeral Noun	NUMNOUN	Numeral as noun	Refers to use of numerals functioning as nouns	I have three.
Object Pronoun	OBJPRO	Object pronoun	Refers to use of pronouns in their objective form	me, us, them, him
Ordinal Determiner	ORDDET	Ordinal as determiner	Refers to use of ordinals functioning as determiners	She took second place.
Ordinal Noun	ORDNOUN	Ordinal as noun	Refers to use of ordinals functioning as nouns	I came first!
General Adverb	OTHERADV	Other adverb	Refers to other adverbs that are not tagged as amplifiers, downtoners, time and place adverbials, etc.	
General Conjunction	OTHRCONJ	Other conjunction	Refers to other conjunctions not tagged as either contrastive or coordinating	
General Interjections	OTHRINTJ	Other interjection	Refers to other interjections that are not tagged as laughter, positive interjection 'Yes', negative interjections 'No'	OMG, WOW!

General Noun	OTHRNOUN	Other noun	Refers to other nouns that are not tagged as numeral, quantifiers, nominalisations, ordinals	
General Subordinator	OTHRSUB	Other subordinator	Refers to other subordinators that are not tagged as time, place, cause, concessive, and conditional subordinators	
General Verb	OTHRVERB	Other verb	Refers to other verbs that are not tagged as private verbs, public verbs, verb-ing, past tense verbs, participle verbs, third person singular, suasive verbs, perception verbs, copular verbs, be as main verb, auxiliary be, auxiliary have, be as main verb, pro-verb do, auxiliary do, stance verb	
Agentless-Passive	PASSIVE	Agentless Passive	Refers to use of passive voice without the inclusion of an agent	He was arrested. She was told not to speak.
By-Passive	PASSVEBY	By passive	Refers to use of passive voice with agent in by clause	He was arrested by the police. She was told not to speak by her teacher.
Past Tense Verbs	PAST	Past tense verbs	Refers to verbs in their past tense form that are not in perfect aspect	went, saved, held
Perception Verbs	PERCEPTVB	Perception verbs	Refers to verbs of perception	hear, smell, taste
Perfect Aspect	PERFECT	Perfect aspect	Refers to any form of HAVE + verb in past participle form	She *had been* to the shops already.
Pied-piping relative	PIEDPIPREL	Pied piping relatives	Refers to the use of preposition + relative pronoun to avoid stranded preposition	with/to whom did Sarah speak?, the box in which it was kept.
Place Adverbs	PLACEADV	Place adverbials	Refers to adverbs indicating place	behind, beneath, downhill
Place Subordinator	PLACESUB	Place subordinators	Refers to subordinators indicating place	I will find you *wherever* you go, you can find me *where* the food table is.
Possessive noun	POSESNOUN	Possessive nouns	Refers to nouns in possessive form	The cat's dinner
Possessive Proper noun	POSESPRPN	Possessive proper nouns	Refers to proper nouns in possessive form	*Donald Trump's* hair
Positive Interjection	POSINTJ	Positive interjections	Refers to any form of YES tagged as an interjection by the Gimpel tagger	Yeahhhh_!, Yup_!, Ya_!
Possessive	POSSDET	Possessive determiners	Refers to determiners which indicate	our cat, your house, their

Determiner			possession	garden, his eyebrows
Possessive Pronoun	POSSPRO	Possessive pronouns	Refers to pronouns which indicate possession	It is ours/mine/yours/theirs/his/hers.
Predicative Adjective	PREDADJ	Predicative adjectives	Refers to adjectives which come after a copular verb	I am *great*! She looks *crazy*, Gyms smell *nasty*.
Pre Determiner	PREDET	Pre-determiners	Refers to determiners which come before determiners	*All* the people in this room are intelligent.
Preposition	PREP	Prepositions	Refers to the use of prepositions	*down* the road, *in* your car
Auxiliary Contraction	PROCONTRACT	Pronoun with contracted verb	Refers to when the verb is contracted with pronoun	I'm, She'd, They've, You'll, That's
Progressive Aspect	PROGRESSIVE	Progressive aspect	Refers to any form of BE plus (up to 2/3 adverbs and) verb ending in -ING	
Quantifier-Pronoun	PROQUAN	Quantifying pronoun (Indefinite pronouns)	Refers to pronouns which indicate quantity or are indefinite pronouns	anything, somebody, I had a *few,* she had *several, some* of the men, *all* of the men
Quantifier-Pronoun-Contraction	CONTRACTPROQUAN	Quantifying pronoun (Indefinite pronouns) with contracted verb	Refers to quantifying pronouns with the verb contracted	noone's happy today, everyone's been before
Quantifier-Pronoun-Possessive	POSESPROQUAN	Possessive Quantifying pronoun (Indefinite pronouns)	Refers to quantifying pronouns in their possessive form	somebody's jumper
Pro-verb Do	PROVDO	Pro-verb DO	Refers to DO used as a main verb	I hate what he has *done*, She *did* it!
Proper Noun	PRPN	Proper noun	Refers to anything tagged as a proper noun	
Private Verb	PRVV	Private verb	Refers to private verbs: used to encode feelings, opinions, emotions, cognition	believe, think, know, learn
Public Verb	PUBV	Public verb	Refers to public verbs: used to report on speech	told, said, shouted
Quantifier-Pre-determiner	QUANPREDET	Quantifier as pre-determiner	Refers to quantifier as a predeterminer	*All* the people in this room are intelligent.
WH-word+BE	QBE	Question BE: BE following WH word	Refers to WH-word + BE	Why are you going?

WH-word+DO	QDO	Question DO: DO following WH word	Refers to WH-word + DO	When do you care?
Question Mark	QUES	Question mark	Refers to the use of question mark	?, !?, !?!
Reflexive Pronoun	REFLEXPRO	Reflexive pronoun	Refers to pronouns in their reflexive form	myself, herself, ourselves, themselves, yourself
Relative-Clause-Obj.Gap	RELCLAUSEN	Relative clause object gap	Refers to relative clause with object gap	The man that the gypsy cursed
Relative-Clause-Subj.Gap	RELCLAUSESUBGAP	Relative clause subject gap	Refers to relative clauses with subject gap	The man that was cursed
Profanity	SWEAR	Various types of swear words	Refers to words that can be used to offend/abuse as well as swear words generally. They may also be used harmlessly	
Third-Person-Singular-Verb	SINFLECT	Third person singular verb ending -s	Refers to verbs ending in -s	thinks, has, takes
Semi-Colon	SMCOLON	Semi colon	Refers to use of semicolon	
Second-Person-Pronoun	SNDPP	Second person pronoun	Refers to pronouns: subject/object/possessive/reflexive and possessive determiners that refer to the second person: singular and plural plus contracted forms	you, yours, you're, your
Split-Infinitive	SPLINFINITIVE	Split infinitive	Refers to verb in infinitive form separated by adverb(s)	to really hate, to not like
Stance Verb	STANCEVB	Stance verb	Refers to verbs used to encode stance	want, seem, appear, like, love, prefer, need …
Suasive Verb	SUAV	Suasive verb	Refers to verbs which refer to persuasion	beg, insist, command, demand, allow…
Subordinating-Clause-Elipted-Subj.	SUBELIPSUBJECT	Subordinator with elipted subject	Refers to subordinators with the subject omitted	the Bible quotes God as saying …
Subject Pronoun	SUBJPRO	Subject pronoun	Refers to pronouns in their subject form	I, she, he, they, we
Superlative	SUPERLATIVE	Superlative	Refers to adjectives and nouns in	the best, the worst, she is

			superlative form	funniest
Synthetic-Negation	SYNNEG	Synthetic negation	Refers to use of nor, neither and no - but not as interjection	No, neither, nor, no more
that-Adjective-Complement	THATADJCOMP	Adjective+that complement clause	Refers to adjective that complement clauses	It's pathetic that you can't sleep without a night light at 40.
that-Noun-Complement	THATNOUNCOMP	Noun+ that complement clause	Refers to noun complement clauses	the fact that you can't sleep without a night light makes you pathetic.
that-Verb-Complement	THATVCOMP	Verb+that complement clause	Refers to PRVV, PUBVB, SUAV + that	I think that you are pathetic for sleeping with a night light.
Third Person Pronoun	THRDPP	Third person pronoun	Refers to pronouns: subject/object/possessive/reflexive and possessive determiners that refer to the third person: singular and plural plus contracted forms	he, she, theirs, themselves, them, hers
Time Adverb	TIMEADV	Time adverb	Refers to adverbs indicating time	I'll be back *soon*
Time Subordinator	TIMESUB	Time subordinator	Refers to subordinators indicating time	*While* his mother slept, he snuck out the window.
Title	TITLE	Title	Refers to titles	Mr. Dr, Miss, Sir
to-Adjective-Complement	TOADJCOMP	Adjective+to complemet clause	Refers to adjective + to complement clause	I am happy to go with Karen.
URL	URL	URL	Refers to URLs: can be meme, gif, status, link to website, video etc.	
Frequency-Adverbs	USUALITY	Adverbs of usuality/frequency	Refers to adverbs indicating how often	always, never, often
Initial Verb-ING	VBIG	Initial verb ing -ing form (probably gerund/nominalisation)	Refers to when verb ending in -ing is the first verb in the Tweet (after initial mentioning) or if it is the first verb after a full stop	
Intial Verb-Modal	VBIMD	Initial verb that is a modal verb (probably a request)	Refers to when modal verb is the first verb in the Tweet (after initial mentioning) or if a modal verb is the first verb after a full stop	
Initial Verb-Ques	VBIQ	Initial verb that is most likely to be a question		

Initial Verb-S	VBIS	Initial verb ending in -s	Refers to when verb ending in -s is the first verb in the Tweet (after initial mentioning) or if it is the first verb after a full stop (except for a select few verbs which can be used for imperatives)	
Initial Verb-Past	VBIX	Initial verb in past tense form	Refers to when verb in past tense/past participle form is the first verb in the Tweet (after initial mentioning) or if it is the first verb after a full stop	
WH-Word	WHW	WH word	Refers to use of WH words	when, why, who, what, how
WH-clause	WHCLAUSE	WH clause	Refers to WH clauses	Do you understand *what cooperation is?*
Initial-Mention	INITIALMENTION	Initial Mention	Refers to Tweet initial mentioning	\@username how are you?
