# Supplementary material for: Stylistic variation on the Donald Trump Twitter account: A linguistic analysis of tweets posted between 2009 and 2018
Source: PLoS One. 2019 Sep 25;14(9):e0222062. doi: 10.1371/journal.pone.0222062 (PMC6760825; doi:10.1371/journal.pone.0222062)
Supplement: S2 File — This file contains information on the set of linguistic features analysed in this study. (ZIP) [file pone.0222062.s002.zip › FEATURE_SET/Trump_Feature _Set.pdf]

**Trump Feature Set**	**Features in > 5% Tweets**		
**TAG**	**Feature**	**Description**	**Example**
AMPLIFIER	Amplifier	Refers to adverbs used to intensify the verb/adjective	very, absolutely, so
ANALNEG	Analytic-Neg	Refers to 'not' plus contracted forms	can't, cannot, not
ATTRIBADJ	Attributive-Adjective	Adjectives that come before the noun and any other adjective not tagged as predicative	The *big* cat
PROCONTRACT	Auxiliary Contraction	Refers to when the verb is contracted with pronoun	I'm, She'd, They've, You'll, That's
AUXDO	Auxiliary-Do	Refers to any form of DO that is followed by (up to three adverbs and) a verb	I *do* not like cheese, I *did* take the bins out
BRACKET	Bracket	Refers to the use of brackets	
CAPS	Capital	Refers to two or more capital letters that is not tagged as an acronym/URL/mentioned username	
COLON	Colon	Refers to the use of colons	:
COMMA	Comma	Refers to the use of commas	,
CNTRSTCONJ	Contrastive Conj.	Refers to conjunctions that signal a contrast is being made	but, by contrast
CCONJ	Coordinating conj.	Refers to coordinating conjunctions	and, &
BEMV	Copula BE	Refers to when BE is the main verb and when BE is in its copular form; that is, when it is followed by a predicative adjective	She *is* a beautiful woman; She *is* beautiful
DEFART	Definite Article	Refers to the use of the definite article	
EXCLAM	Exclamation Mark	Refers to the use of exclamation marks	!!!, !?
FSTPP	First Pers. Pron.	Refers to pronouns: subject/object/possessive/reflexive and possessive determiners that refer to the first person: singular and plural plus contracted forms	I, We, us, me, myself, ourselves, ours, our, my, mine
OTHERADV	General Adverb	Refers to other adverbs that are not tagged as amplifiers, downtoners, time and place adverbials, etc.	
OTHRINTJ	General	Refers to other interjections that are not tagged as laughter, positive	OMG, WOW!

	Interjections	interjection 'Yes', negative interjections 'No'	
OTHRNOUN	General Noun	Refers to other nouns that are not tagged as numeral, quantifiers, nominalisations, ordinals	
OTHRVERB	General Verb	Refers to other verbs that are not tagged as private verbs, public verbs, verb-ing, past tense verbs, participle verbs, third person singular, suasive verbs, perception verbs, copular verbs, be as main verb, auxiliary be, auxiliary have, be as main verb, pro-verb do, auxiliary do, stance verb	
GERUND	Gerund	Refers to prepositional complement: when a preposition is followed by noun in -ing form (but this is tagged by Gimpel tagger as a verb)	Sarah talked about *leaving* her job
HASHTAG	Hashtag	Refers to the use of Hashtag	
HAVEMV	Have Main Verb	Refers to when any form of HAVE is the main verb	She *has* so much money, I *had* seven chocolates
IMPERATIVE	Imperative	Refers to clauses in imperative mood	Go away!, Don't be foolish!
INDEFART	Indefinite Article	Refers to use of indefinite article	a, an
INFINITIVE	Infinitive	Refers to verbs in infinistive form that is not adjective + to complement clause or split infinitive	to be, to have
INITIALMENTION	Initial Mention	Refers to Tweet initial mentioning	\@username how are you?
MDNEC	Modal of Necessity	Refers to modals indicating necessity/obligation	should, mustn't, ought
MDPOSS	Modal of Possibility	Refers to modals indicating probability/possibility/ability	can, may, mightn't
MDPRED+GOINGTOBE	Modal of Prediction	Refers to modals indicating prediction and the BE-going-to construction	will, shall, I'll, I'm going to
NOMIN	Nominalisation	Refers to when verbs/adjectives are converted into nouns	action, statement
MENTION	Non-initial Ment.	Refers to mentioning that is not initial	I know that \@username likes cheese
NUMDET	Numeral Determiner	Refers to use of numerals functioning as determiners	Three dogs
NUMNOUN	Numeral Noun	Refers to use of numerals functioning as nouns	I have three.
OBJPRO	Object Pronoun	Refers to use of pronouns in their objective form	me, us, them, him
PASSIVE	Passive-Agentless	Refers to use of passive voice without the inclusion of an agent	He was arrested. She was told not to speak.

PAST	Past Tense Verbs	Refers to verbs in their past tense form that are not in perfect aspect	went, saved, held
PERCEPTVB	Perception Verbs	Refers to verbs of perception	hear, smell, taste
PERFECT	Perfect Aspect	Refers to any form of HAVE + verb in past participle form	She *had been* to the shops already.
FULSTOP	Period	Refers to the use of full stop	.
MULTIWVB	Phrasal verb	Refers to both prepositional and particle verbs	catch up, check up
POSSDET	Possessive Determiner	Refers to determiners which indicate possession	our cat, your house, their garden, his eyebrows
POSESPRPN	Possessive Proper noun	Refers to proper nouns in possessive form	*Donald Trump's* hair
PREDADJ	Predicative Adjective	Refers to adjectives which come after a copular verb	I am *great*! She looks *crazy*, Gyms smell *nasty*.
PREP	Preposition	Refers to the use of prepositions	*down* the road, *in* your car
PRVV	Private Verb	Refers to private verbs: used to encode feelings, opinions, emotions, cognition	believe, think, know, learn
PROVDO	Pro-verb Do	Refers to DO used as a main verb	I hate what he has *done*, She *did* it!
PROGRESSIVE	Progressive Aspect	Refers to any form of BE plus (up to 2/3 adverbs and) verb ending in -ING	
IT	Pronoun IT	Refers to any form of pronoun IT: contracted, reflexive, possessive and possessive determiner	It is great, it's okay, itself, its
PRPN	Proper Noun	Refers to anything tagged as a proper noun	
PUBV	Public Verb	Refers to public verbs: used to report on speech	told, said, shouted
DETQUAN	Quantifier-Determiner	Refers to the use of this, that, these, those as a determiner; that is followed by noun	
PROQUAN	Quantifier-Pronoun	Refers to pronouns which indicate quantity or are indefinite pronouns	anything, somebody, I had a *few,* she had *several, some* of the men, *all* of the men
QUES	Question Mark	Refers to the use of question mark	?, !?, !?!

RELCLAUSESUBGAP	Relative-Clause-Subj.Gap	Refers to relative clauses with subject gap	The man that was cursed
SNDPP	Second-Person-Pronoun	Refers to pronouns: subject/object/possessive/reflexive and possessive determiners that refer to the second person: singular and plural plus contracted forms	you, yours, you're, your
STANCEVB	Stance Verb	Refers to verbs used to encode stance	want, seem, appear, like, love, prefer, need …
SUBJPRO	Subject Pronoun	Refers to pronouns in their subject form	I, she, he, they, we
SUPERLATIVE	Superlative	Refers to adjectives and nouns in superlative form	the best, the worst, she is funniest
THRDPP	Third Person Pronoun	Refers to pronouns: subject/object/possessive/reflexive and possessive determiners that refer to the third person: singular and plural plus contracted forms	he, she, theirs, themselves, them, hers
SINFLECT	Third-Person-Singular-Verb	Refers to verbs ending in -s	thinks, has, takes
TIMEADV	Time Adverb	Refers to adverbs indicating time	I'll be back *soon*
URL	URL	Refers to URLs: can be meme, gif, status, link to website, video etc.	
WHW	WH-Word	Refers to use of WH words	when, why, who, what, how
