# Supplementary figures and images for: Stylistic variation on the Donald Trump Twitter account: A linguistic analysis of tweets posted between 2009 and 2018
Source: PLoS One. 2019 Sep 25;14(9):e0222062. doi: 10.1371/journal.pone.0222062 (PMC6760825; doi:10.1371/journal.pone.0222062)

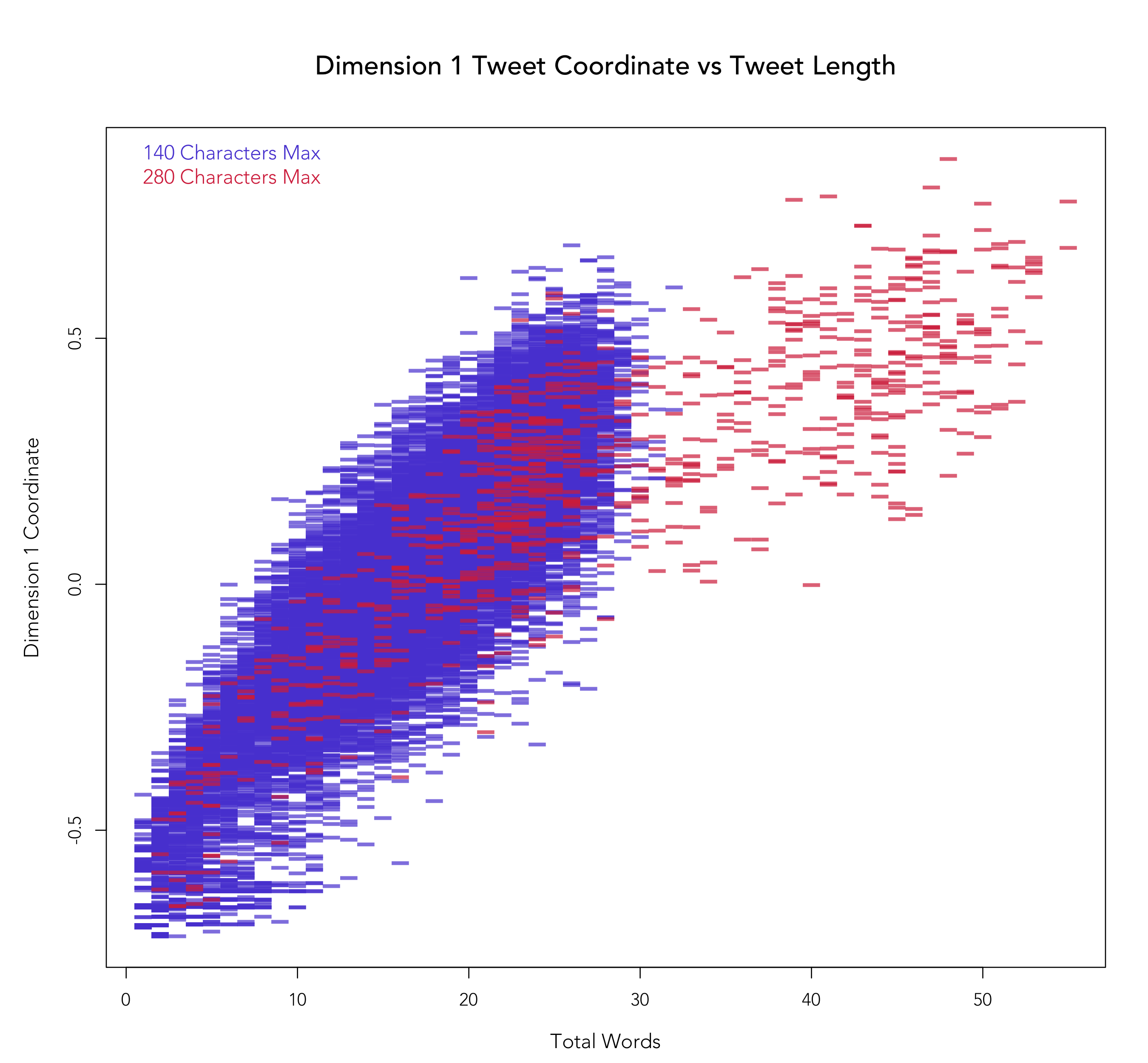

Supplement: S3 File — This file contains the full dataset, R code, and output for the quantitative analysis conducted for this study. (ZIP) [file pone.0222062.s003.zip › R_ANALYSIS/FIG/Fig6.tiff]

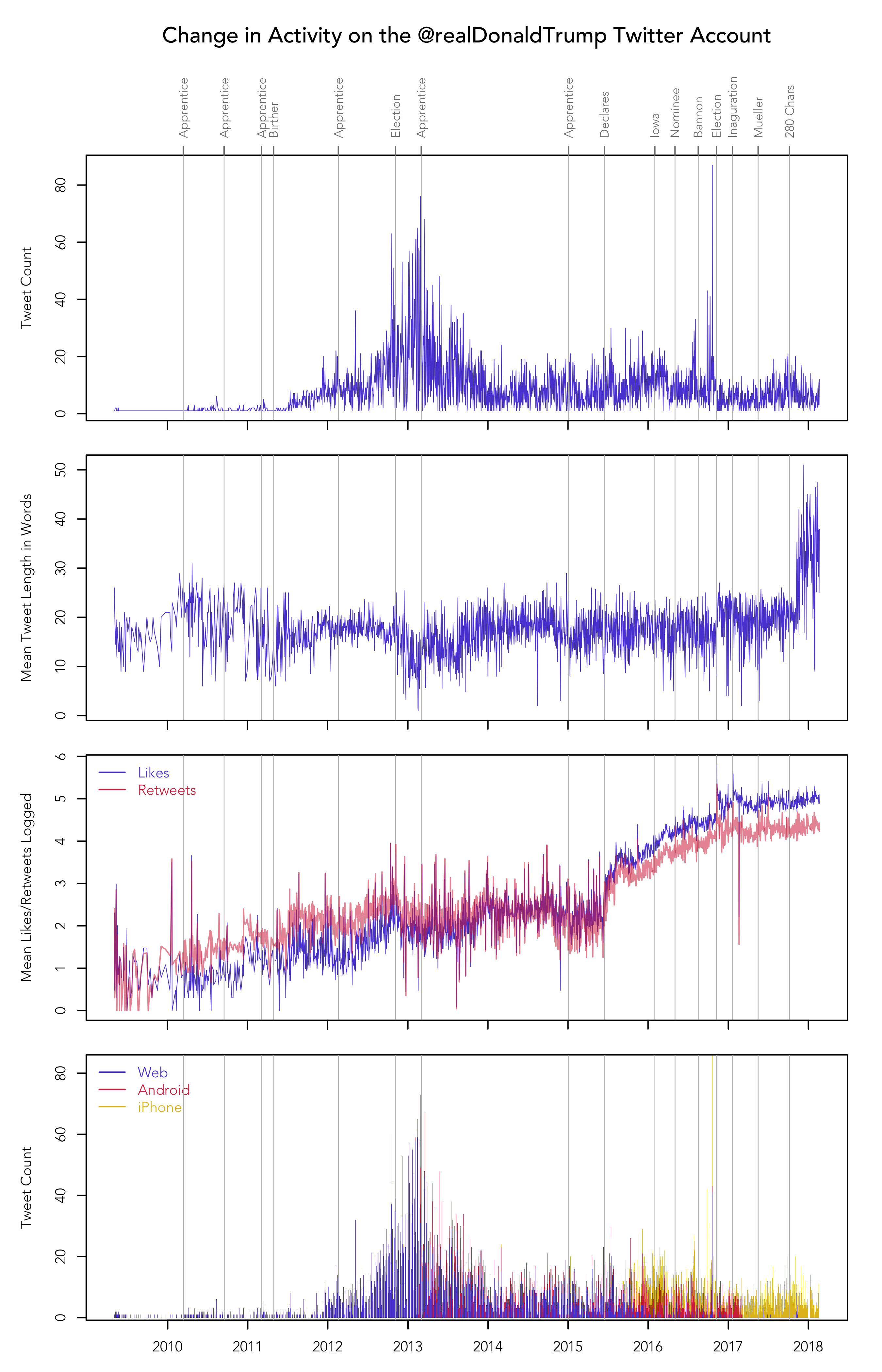

Supplement: S3 File — This file contains the full dataset, R code, and output for the quantitative analysis conducted for this study. (ZIP) [file pone.0222062.s003.zip › R_ANALYSIS/FIG/Fig1.tiff]

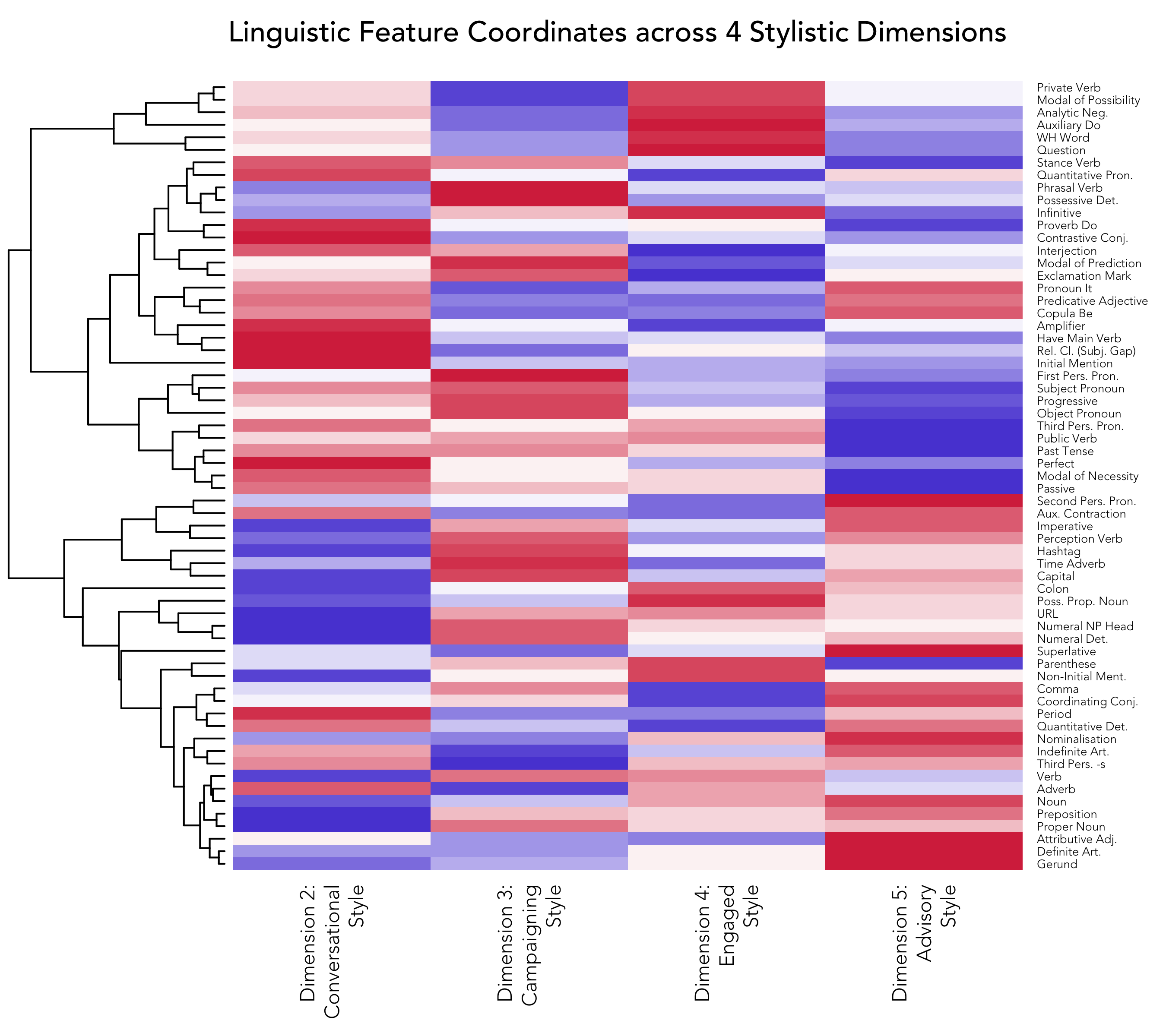

Supplement: S3 File — This file contains the full dataset, R code, and output for the quantitative analysis conducted for this study. (ZIP) [file pone.0222062.s003.zip › R_ANALYSIS/FIG/Fig3.tiff]

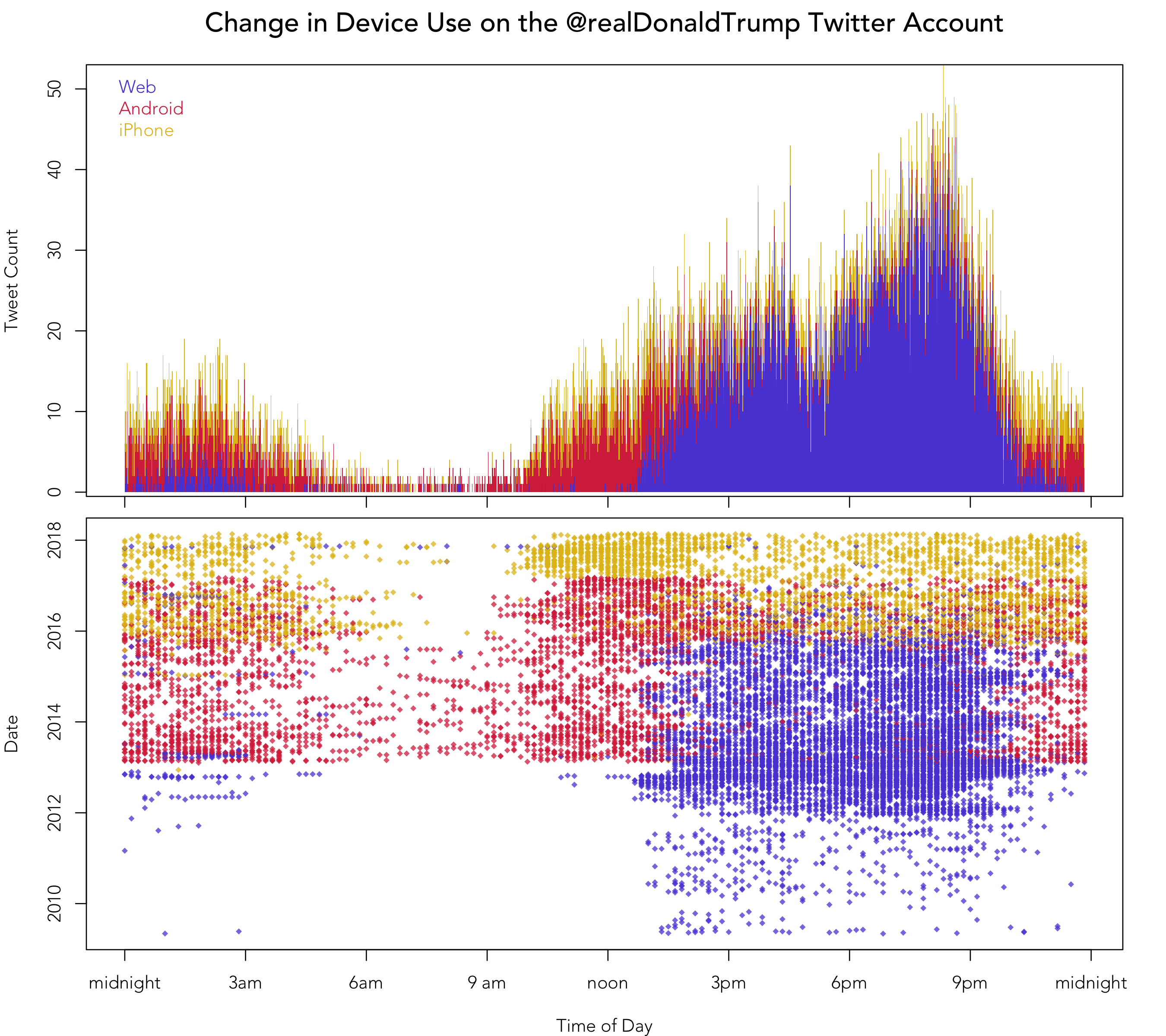

Supplement: S3 File — This file contains the full dataset, R code, and output for the quantitative analysis conducted for this study. (ZIP) [file pone.0222062.s003.zip › R_ANALYSIS/FIG/Fig2.tiff]

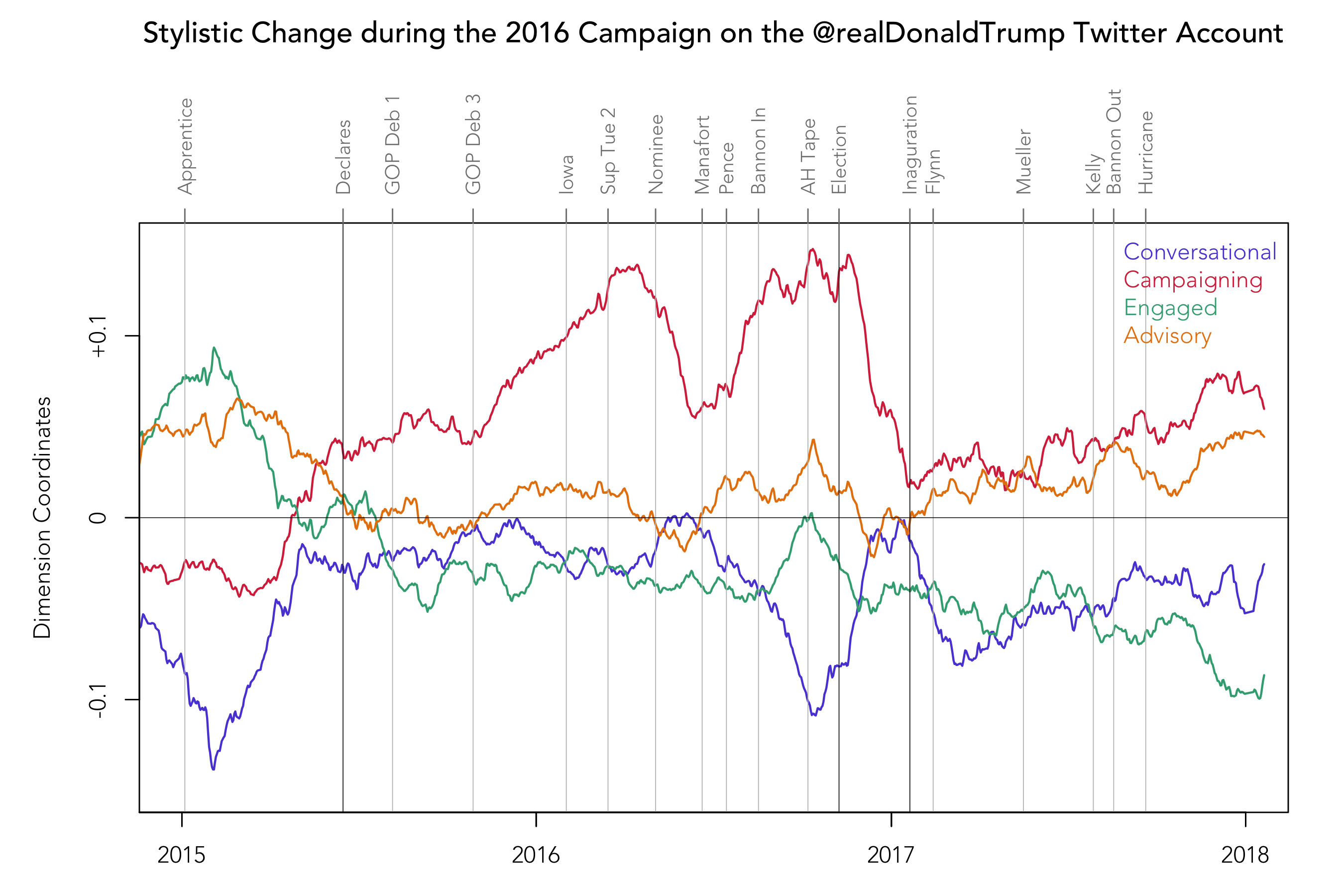

Supplement: S3 File — This file contains the full dataset, R code, and output for the quantitative analysis conducted for this study. (ZIP) [file pone.0222062.s003.zip › R_ANALYSIS/FIG/Fig5.tiff]

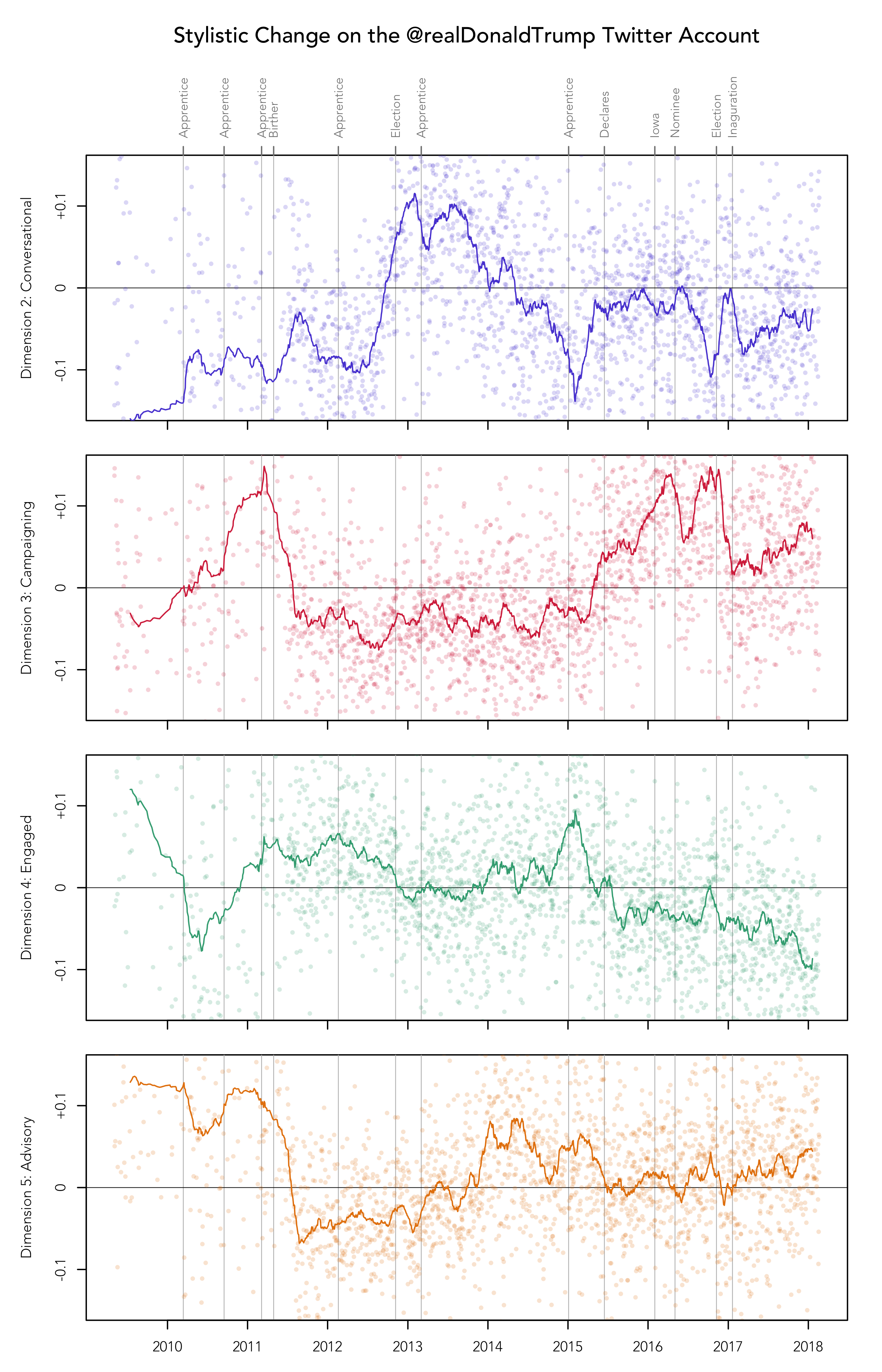

Supplement: S3 File — This file contains the full dataset, R code, and output for the quantitative analysis conducted for this study. (ZIP) [file pone.0222062.s003.zip › R_ANALYSIS/FIG/Fig4.tiff]
