# Supplementary material for: Stylistic variation on the Donald Trump Twitter account: A linguistic analysis of tweets posted between 2009 and 2018
Source: PLoS One. 2019 Sep 25;14(9):e0222062. doi: 10.1371/journal.pone.0222062 (PMC6760825; doi:10.1371/journal.pone.0222062)
Supplement: S3 File — This file contains the full dataset, R code, and output for the quantitative analysis conducted for this study. (ZIP) [file pone.0222062.s003.zip › R_ANALYSIS/TRUMP_FINAL.html]

Quantitative Stylistic Analysis of the Trump Twitter Account in R


# Quantitative Stylistic Analysis of the Trump Twitter Account in R

### R Markdown for ‘Isobelle Clarke and Jack Grieve. 2019. Stylistic variation on the Donald Trump Twitter account: A linguistic analysis of tweets posted between 2009 and 2018. Accepted for publication in PloS one.’

#### Jack Grieve (j.grieve@bham.ac.uk)

#### 2019-08-15

# 1 Introduction

This markdown is part of the repository/supplemental materials for Clarke & Grieve (2019). It presents the R code and the results for the quantitative stylistic analysis of the Donald Trump Twitter for that paper. This includes the code for conducting the statistical analysis and for plotting the figures presented in the paper, as well as some additional analyses. This markdown, as well as the data files and additional materials, are available in the supplementary materials for this paper hosted by PLoS one and in our OSF repository: https://osf.io/qhm5z/.

## 1.1 Packages

We use the following packages.

```
library(dplyr)
```

```
## 
## Attaching package: 'dplyr'
```

```
## The following objects are masked from 'package:stats':
## 
##     filter, lag
```

```
## The following objects are masked from 'package:base':
## 
##     intersect, setdiff, setequal, union
```

```
library(ggplot2)
library(FactoMineR)
library(factoextra)
```

```
## Welcome! Related Books: `Practical Guide To Cluster Analysis in R` at https://goo.gl/13EFCZ
```

```
library(forecast)
```

## 1.2 Aesthetics

We also specify some aesthetic settings for fonts and colour palettes used in the figures.

```
quartzFonts(avenir = c("Avenir Light", 
                       "Avenir Medium", 
                       "Avenir Light Oblique",
                       "Avenir Medium Oblique"))

bluec <- rgb(90, 75, 215, maxColorValue=255)
redc <- rgb(215, 50, 75, maxColorValue=255)
yellowc <- rgb(225, 190, 30, maxColorValue=255)
orangec <- rgb(230, 130, 10, maxColorValue=255)
greenc <- rgb(60, 170, 130, maxColorValue=255)
```

# 2 Data

We downloaded all posts from the @realDonaldTrump Twitter account from the Trump Twitter Archive (http://www.trumptwitterarchive.com). Our corpus, which is included in the supplementary materials, consists of all Tweets except retweets posted on the @realDonaldTrump Twitter account between 2009-05-04 and 2018-02-20. We then tagged the corpus using the Gimpel Tagger (https://www.aclweb.org/anthology/P11-2008) and identified a wide range of grammatical features using perl scripts. Based on this linguistic analysis we produced a dataset consisting of the presence or absence of 63 linguistic variables across 21,739 tweets, which is available with this repo. We focus on presence and absence, as opposed to frequencies, because tweets are so short.

We begin the quantitative analysis by reading in this dataset and ordering it by date. In addition to the 63 categorical linguistic variables, which are stored in columns 3 to 65, columns 1 and 2 contain the unique Twitter ID of each tweet and the total number of words in each tweet, and columns 66 to 71 contain metadata: date, time period (which we coded based on date), time of day, source, retweet count, and like count.

```
tweets <- read.table("TRUMP_DATA.txt", header = TRUE, sep = ",")
dim(tweets)
```

```
## [1] 21739    71
```

```
tweets <- tweets[order(tweets$DATE), ]
head(tweets$DATE)
```

```
## [1] 2009-05-04 2009-05-05 2009-05-08 2009-05-08 2009-05-12 2009-05-12
## 2399 Levels: 2009-05-04 2009-05-05 2009-05-08 2009-05-12 ... 2018-02-20
```

```
tail(tweets$DATE)
```

```
## [1] 2018-02-20 2018-02-20 2018-02-20 2018-02-20 2018-02-20 2018-02-20
## 2399 Levels: 2009-05-04 2009-05-05 2009-05-08 2009-05-12 ... 2018-02-20
```

```
summary(tweets)
```

```
##     TWEETID        WORDCOUNT     AMPLIFIER ANALNEG   ATTRIBADJ AUXDO    
##  Min.   :    1   Min.   : 1.00   A:18817   A:18539   A: 7632   A:20455  
##  1st Qu.: 5436   1st Qu.:12.00   P: 2922   P: 3200   P:14107   P: 1284  
##  Median :10870   Median :18.00                                          
##  Mean   :10870   Mean   :16.76                                          
##  3rd Qu.:16304   3rd Qu.:22.00                                          
##  Max.   :21739   Max.   :55.00                                          
##                                                                         
##  BEMV      BRACKET   CAPS      CCONJ     CNTRSTCONJ COLON     COMMA    
##  A:14233   A:20526   A:16595   A:14227   A:20566    A:19922   A:14175  
##  P: 7506   P: 1213   P: 5144   P: 7512   P: 1173    P: 1817   P: 7564  
##                                                                        
##                                                                        
##                                                                        
##                                                                        
##                                                                        
##  DEFART    DETQUAN   EXCLAM    FSTPP     FULSTOP   GERUND    HASHTAG  
##  A:12004   A:20101   A:12744   A:13523   A: 7619   A:20518   A:19120  
##  P: 9735   P: 1638   P: 8995   P: 8216   P:14120   P: 1221   P: 2619  
##                                                                       
##                                                                       
##                                                                       
##                                                                       
##                                                                       
##  HAVEMV    IMPERATIVE INDEFART  INFINITIVE IT        MDNEC     MDPOSS   
##  A:20307   A:18902    A:15684   A:17907    A:19051   A:20399   A:20311  
##  P: 1432   P: 2837    P: 6055   P: 3832    P: 2688   P: 1340   P: 1428  
##                                                                         
##                                                                         
##                                                                         
##                                                                         
##                                                                         
##  MDPRED    MENTION   MULTIWVB  NOMIN     NUMDET    NUMNOUN   OBJPRO   
##  A:17642   A:15255   A:19605   A:17116   A:19154   A:19257   A:19511  
##  P: 4097   P: 6484   P: 2134   P: 4623   P: 2585   P: 2482   P: 2228  
##                                                                       
##                                                                       
##                                                                       
##                                                                       
##                                                                       
##  OTHERADV  OTHRINTJ  OTHRNOUN  OTHRVERB  PASSIVE   PAST      PERCEPTVB
##  A:15045   A:18945   A: 3369   A: 6994   A:20212   A:14833   A:20349  
##  P: 6694   P: 2794   P:18370   P:14745   P: 1527   P: 6906   P: 1390  
##                                                                       
##                                                                       
##                                                                       
##                                                                       
##                                                                       
##  PERFECT   POSESPRPN POSSDET   PREDADJ   PREP      PROCONTRACT PROGRESSIVE
##  A:20058   A:20192   A:16609   A:18020   A: 5589   A:20347     A:18951    
##  P: 1681   P: 1547   P: 5130   P: 3719   P:16150   P: 1392     P: 2788    
##                                                                           
##                                                                           
##                                                                           
##                                                                           
##                                                                           
##  PROQUAN   PROVDO    PRPN      PRVV      PUBV      QUES     
##  A:20058   A:20341   A: 6565   A:19141   A:18674   A:20343  
##  P: 1681   P: 1398   P:15174   P: 2598   P: 3065   P: 1396  
##                                                             
##                                                             
##                                                             
##                                                             
##                                                             
##  RELCLAUSESUBGAP SINFLECT  SNDPP     STANCEVB  SUBJPRO   SUPERLATIVE
##  A:20602         A:12866   A:17997   A:20063   A:13914   A:20577    
##  P: 1137         P: 8873   P: 3742   P: 1676   P: 7825   P: 1162    
##                                                                     
##                                                                     
##                                                                     
##                                                                     
##                                                                     
##  THRDPP    TIMEADV   URL       WHW       INITIALMENTION         DATE      
##  A:16945   A:17738   A:15207   A:19508   A:19099        2016-10-20:   87  
##  P: 4794   P: 4001   P: 6532   P: 2231   P: 2640        2013-02-27:   76  
##                                                         2013-03-19:   68  
##                                                         2013-02-13:   65  
##                                                         2012-10-17:   63  
##                                                         2013-02-06:   61  
##                                                         (Other)   :21319  
##               JOB              TIME              SOURCE     
##  CAMPAIGN       : 5142   20:40:19:    6   Web Client:11252  
##  PRE-CAMPAIGN   :13852   18:59:31:    5   Android   : 4659  
##  PRESIDENT      : 2392   19:25:03:    5   iPhone    : 4224  
##  PRESIDENT-ELECT:  353   19:39:29:    5   TweetDeck :  483  
##                          20:04:45:    5   TwitLonger:  404  
##                          20:28:22:    5   Instagram :  133  
##                          (Other) :21708   (Other)   :  584  
##     RETWEET            FAV        
##  Min.   :     0   Min.   :     0  
##  1st Qu.:    36   1st Qu.:    22  
##  Median :   277   Median :   178  
##  Mean   :  3990   Mean   : 14236  
##  3rd Qu.:  3356   3rd Qu.:  8812  
##  Max.   :369530   Max.   :633253  
##
```

## 2.1 Descriptives

# 3 Tweet Length

We measure tweet length (in words) across the entire corpus, as well as before and after the character limit of Twitter was raised from 140 to 280 characters.

```
summary(tweets$WORDCOUNT)
```

```
##    Min. 1st Qu.  Median    Mean 3rd Qu.    Max. 
##    1.00   12.00   18.00   16.76   22.00   55.00
```

```
summary(tweets$WORDCOUNT[1:21234])              # Before 280 Character Limit
```

```
##    Min. 1st Qu.  Median    Mean 3rd Qu.    Max. 
##    1.00   12.00   18.00   16.39   22.00   53.00
```

```
summary(tweets$WORDCOUNT[21235:nrow(tweets)])   # After 280 Character Limit
```

```
##    Min. 1st Qu.  Median    Mean 3rd Qu.    Max. 
##    2.00   22.00   35.00   32.61   45.00   55.00
```

We find a small inverse correlation between mean tweet length per day and the number of tweets sent per day.

```
wc <- aggregate(WORDCOUNT ~ DATE, tweets, mean)
tc <- count(tweets,DATE)
cor(tc$n, wc$WORDCOUNT)
```

```
## [1] -0.2315537
```

```
plot(tc$n, wc$WORDCOUNT)
```

## 3.1 Sources

We also compare tweet counts by sources. Most tweets came from web browsers or smart phones.

```
summary(tweets$SOURCE )
```

```
##        Android     BlackBerry       Facebook      Instagram           iPad 
##           4659             96            105            133             48 
##         iPhone   Media Studio  Mobile Web M5         Neatly      Periscope 
##           4224            126             55              5              7 
##      TweetDeck     Twitlonger     TwitLonger    Twitter Ads Twitter Mirror 
##            483             23            404             97              1 
##  Twitter QandA           Vine     Web Client       Websites 
##             10             10          11252              1
```

```
sum(tweets$SOURCE == "Web Client")/nrow(tweets)
```

```
## [1] 0.5175951
```

```
sum(tweets$SOURCE == "Android")/nrow(tweets)
```

```
## [1] 0.2143153
```

```
sum(tweets$SOURCE == "iPhone")/nrow(tweets)
```

```
## [1] 0.1943052
```

```
sum(tweets$SOURCE == "TweetDeck")/nrow(tweets)
```

```
## [1] 0.02221813
```

```
(sum(tweets$SOURCE == "Web Client") +
    sum(tweets$SOURCE == "Android") +
    sum(tweets$SOURCE == "iPhone"))/
  nrow(tweets)
```

```
## [1] 0.9262156
```

```
pie(table(tweets$SOURCE))
```

## 3.2 First Time Series Graphing Function

Next, we make a graphing function to generate time series charts for the meta data, with important dates marked on the x-axis.

```
tsgraph <- function(day, val, color, ylabel){
  plot(day, val, 
       col = color,
       type= "l", 
       lwd=.5,
       axes = FALSE,
       frame.plot=TRUE,
       xlab= "Date",
       ylab= ylabel)
  abline(v=as.Date("2010-03-14"), col="grey",lwd=.6)   # Apprentice 
    abline(v=as.Date("2011-03-06"), col="grey",lwd=.6) # Apprentice 
    abline(v=as.Date("2012-02-19"), col="grey",lwd=.6) # Apprentice 
    abline(v=as.Date("2013-03-03"), col="grey",lwd=.6) # Apprentice 
    abline(v=as.Date("2015-01-04"), col="grey",lwd=.6) # Apprentice 
    abline(v=as.Date("2010-09-16"), col="grey",lwd=.6) # Apprentice
    abline(v=as.Date("2011-04-30"), col="grey",lwd=.6) # Birther Attacks
    abline(v=as.Date("2012-11-06"), col="grey",lwd=.6) # 2012 Election
    abline(v=as.Date("2015-06-16"), col="grey",lwd=.6) # Announces
    abline(v=as.Date("2016-02-01"), col="grey",lwd=.6) # Iowa
    abline(v=as.Date("2016-05-03"), col="grey",lwd=.6) # Presumptive Nominee
    abline(v=as.Date("2016-08-17"), col="grey",lwd=.6) # Bannon
    abline(v=as.Date("2016-11-08"), col="grey",lwd=.6) # Election
    abline(v=as.Date("2017-01-20"), col="grey",lwd=.6) # Inauguration
    abline(v=as.Date("2017-05-17"), col="grey",lwd=.6) # Mueller
    abline(v=as.Date("2017-10-07"), col="grey",lwd=.6) # Twitter Length Increase
  axis(side=2, tick = TRUE)
}
```

## 3.3 Generating Figure 1

We use this graphing function to generate Figure 1 for the paper, which presents four aligned time series from 2009-2018 together on one grid. These time series show total tweets by day, mean tweet length by day, mean likes and retweets by day, and total tweets by device by day.

```
tiff("FIG/Fig1.tiff", width=2700, height=4200, res=400)

# General Parameters

  par(mfrow=c(4,1),
      oma=c(2, 0, 8, 0),
      family="avenir")

# Panel 1: Tweets by Day

  par(mar=c(1, 5, 1, 2))
  temp <- count(tweets,DATE)
  tsgraph(day = as.Date(temp$DATE),
          val = temp$n,
          color = bluec,
          ylabel= "Tweet Count")
  axis(side=3, tick = TRUE,  las=2, cex.axis=.85, outer=FALSE,
       col.ticks = "gray50", 
       col.axis = "gray50", col=NA,
       at=c(as.Date("2010-03-14"),
            as.Date("2010-09-16"),
            as.Date("2011-03-06"),
            as.Date("2011-04-30"), 
            as.Date("2012-02-19"),
            as.Date("2012-11-06"), 
            as.Date("2013-03-03"), 
            as.Date("2015-01-04"),
            as.Date("2015-06-16"), 
            as.Date("2016-02-01"), 
            as.Date("2016-05-03"), 
            as.Date("2016-08-17"), 
            as.Date("2016-11-08"),
            as.Date("2017-01-20"),
            as.Date("2017-05-17"),
            as.Date("2017-10-07")),
       labels=c("Apprentice", "Apprentice", "Apprentice", 
                "Birther", "Apprentice", "Election",  
                "Apprentice", "Apprentice", "Declares", 
                "Iowa", "Nominee", "Bannon",
                "Election", "Inaguration","Mueller",
                "280 Chars"))
  mtext(text=expression(bold("Change in Activity on the @realDonaldTrump Twitter Account")), 
        side=3, line=6, las=1, cex=1)
  axis(side=1, tick = TRUE, cex.axis=1, 
       labels= FALSE,
       at=c(as.Date("2010-01-01"),
            as.Date("2011-01-01"), 
            as.Date("2012-01-01"), 
            as.Date("2013-01-01"), 
            as.Date("2014-01-01"), 
            as.Date("2015-01-01"), 
            as.Date("2016-01-01"), 
            as.Date("2017-01-01"), 
            as.Date("2018-01-01")))

# Panel 2: Mean Tweet Length by Day

  par(mar=c(1, 5, 1, 2))
  temp <- aggregate(WORDCOUNT ~ DATE, tweets, mean)
  tsgraph(day = as.Date(temp$DATE),
          val = temp$WORDCOUNT,
          color = bluec,
          ylabel = "Mean Tweet Length in Words")
  axis(side = 1, tick = TRUE, cex.axis = 1, 
       labels = FALSE,
       at=c(as.Date("2010-01-01"),
            as.Date("2011-01-01"), 
            as.Date("2012-01-01"), 
            as.Date("2013-01-01"), 
            as.Date("2014-01-01"), 
            as.Date("2015-01-01"), 
            as.Date("2016-01-01"), 
            as.Date("2017-01-01"), 
            as.Date("2018-01-01")))

# Panel 3: Like/Retweets by Day

  par(mar=c(1, 5, 1, 2))
  temp <- aggregate(FAV ~ DATE, tweets, mean)
  tsgraph(day = as.Date(temp$DATE),
          val = log10(temp$FAV),
          color = bluec,
          ylabel= "Mean Likes/Retweets Logged")
  temp <- aggregate(RETWEET ~ DATE, tweets, mean)
  lines(as.Date(temp$DATE), log10(temp$RETWEET), 
        col=adjustcolor(redc, alpha.f = 0.55))
  legend("topleft", 
         legend = c("Likes", "Retweets"), 
         lty = c(1,1), 
         lwd=c(1,1), 
         col = c(bluec, redc), 
         text.col = c(bluec, redc), 
         bty="n",
         cex=1)
  axis(side=1, tick = TRUE, cex.axis=1, 
       labels= FALSE,
       at=c(as.Date("2010-01-01"),
            as.Date("2011-01-01"), 
            as.Date("2012-01-01"), 
            as.Date("2013-01-01"), 
            as.Date("2014-01-01"), 
            as.Date("2015-01-01"), 
            as.Date("2016-01-01"), 
            as.Date("2017-01-01"), 
            as.Date("2018-01-01")))

# Panel 4: Device by Day

  temp<-c()
  temp$DATE <- seq.Date(min(as.Date(tweets$DATE)),
                        max(as.Date(tweets$DATE)),
                        by=1)
  temp <- as.data.frame(temp)
  temp.w <- as.data.frame(count(tweets[tweets$SOURCE=="Web Client",],DATE))
    temp.w$DATE <- as.Date(temp.w$DATE)
    temp.w <- merge(temp,temp.w,by="DATE", all.x=TRUE)
    temp.w[is.na(temp.w)] <- 0
  temp.a <- as.data.frame(count(tweets[tweets$SOURCE=="Android",],DATE))
    temp.a$DATE <- as.Date(temp.a$DATE)
    temp.a <- merge(temp,temp.a,by="DATE", all.x=TRUE)
    temp.a[is.na(temp.a)] <- 0
  temp.i <- as.data.frame(count(tweets[tweets$SOURCE=="iPhone",],DATE))
    temp.i$DATE <- as.Date(temp.i$DATE)
    temp.i <- merge(temp,temp.i,by="DATE", all.x=TRUE)
    temp.i[is.na(temp.i)] <- 0
  stackb <- c()
    stackb$W <- temp.w$n
    stackb$A <- temp.a$n
    stackb$I <- temp.i$n
    stackb <- as.data.frame(stackb, row.names=temp.w$DATE)
  par(mar=c(1, 5, 1, 2))
  barplot(t(stackb),
          border=NA,
          col=c(bluec, redc, yellowc),
          space=0,
          axes=FALSE,
          axisnames = FALSE,
          ylab="Tweet Count")
  box()
  legend("topleft", 
         legend = c("Web", "Android", "iPhone"), 
         lty = c(1,1), 
         lwd=c(1,1), 
         col = c(bluec, redc ,yellowc), 
         text.col = c(bluec, redc, yellowc), 
         bty="n",
         cex=1)
  axis(side=2, tick = TRUE)
  axis(side=1, tick = TRUE, cex.axis=1, 
       at=c(which(rownames(stackb) %in% "2010-01-01"),
            which(rownames(stackb) %in% "2011-01-01"), 
            which(rownames(stackb) %in% "2012-01-01"), 
            which(rownames(stackb) %in% "2013-01-01"), 
            which(rownames(stackb) %in% "2014-01-01"), 
            which(rownames(stackb) %in% "2015-01-01"), 
            which(rownames(stackb) %in% "2016-01-01"), 
            which(rownames(stackb) %in% "2017-01-01"), 
            which(rownames(stackb) %in% "2018-01-01")),
       labels=c("2010", "2011", "2012", 
                "2013", "2014", "2015", 
                "2016","2017", "2018"))
  abline(v=which(rownames(stackb) %in% "2010-09-16"), 
         col="grey",lwd=.6)   # Apprentice
  abline(v=which(rownames(stackb) %in% "2011-04-30"), 
         col="grey",lwd=.6) # Birther Attacks
  abline(v=which(rownames(stackb) %in% "2012-11-06"), 
         col="grey",lwd=.6) # 2012 Election
  abline(v=which(rownames(stackb) %in% "2015-06-16"), 
         col="grey",lwd=.6) # Announces
  abline(v=which(rownames(stackb) %in% "2016-02-01"), 
         col="grey",lwd=.6) # Iowa
  abline(v=which(rownames(stackb) %in% "2016-05-03"), 
         col="grey",lwd=.6) # Presumptive Nominee
  abline(v=which(rownames(stackb) %in% "2016-08-17"), 
         col="grey",lwd=.6) # Bannon
  abline(v=which(rownames(stackb) %in% "2016-11-08"), 
         col="grey",lwd=.6) # Election
  abline(v=which(rownames(stackb) %in% "2017-01-20"), 
         col="grey",lwd=.6) # Inauguration
  abline(v=which(rownames(stackb) %in% "2017-05-17"), 
         col="grey",lwd=.6) # Mueller
  abline(v=which(rownames(stackb) %in% "2017-10-07"), 
         col="grey",lwd=.6) # 280 char     
  abline(v=which(rownames(stackb) %in% "2010-03-14"), 
         col="grey",lwd=.6) # Apprentice 
  abline(v=which(rownames(stackb) %in% "2011-03-06"), 
         col="grey",lwd=.6) # Apprentice 
  abline(v=which(rownames(stackb) %in% "2012-02-19"), 
         col="grey",lwd=.6) # Apprentice 
  abline(v=which(rownames(stackb) %in% "2013-03-03"), 
         col="grey",lwd=.6) # Apprentice 
  abline(v=which(rownames(stackb) %in% "2015-01-04"), 
         col="grey",lwd=.6) # Apprentice 
     
dev.off()
```

```
## quartz_off_screen 
##                 2
```

Figure 1

## 3.4 Time of Day Analysis

We also analyse posting patterns by time of day.

First, we covert the time data from being measured by the second to being measured by the minute.

```
tweets$TIME_SHORT <- gsub("(\\d\\d):(\\d\\d):(\\d\\d)","\\1:\\2", tweets$TIME)
```

Second, we make a blank data frame consisting of all the minutes in a day as rows.

```
temp<-c()
temp$TIME_SHORT <- seq.POSIXt(as.POSIXct("00:00", format="%H:%M"),
                              as.POSIXct("23:59", format="%H:%M"),
                              by=60)
temp <- as.data.frame(temp)
temp$TIME_SHORT <- gsub(
  "\\d\\d\\d\\d-\\d\\d-\\d\\d (\\d\\d):(\\d\\d):(\\d\\d)",
  "\\1:\\2", 
  temp$TIME_SHORT)
```

Third, we count all occurences of the three main sources (web client, android, iphone) by minute across all days in the dataset and then merge these counts with the blank minute-by-minute data frame to get complete and comparable counts for each source over the minutes of the day.

```
temp.w <- as.data.frame(count(tweets[tweets$SOURCE=="Web Client",],TIME_SHORT))
temp.w <- merge(temp, temp.w, by="TIME_SHORT", all.x=TRUE)
temp.w[is.na(temp.w)] <- 0

temp.a <- as.data.frame(count(tweets[tweets$SOURCE=="Android",],TIME_SHORT))
temp.a <- merge(temp,temp.a,by="TIME_SHORT", all.x=TRUE)
temp.a[is.na(temp.a)] <- 0

temp.i <- as.data.frame(count(tweets[tweets$SOURCE=="iPhone",],TIME_SHORT))
temp.i <- merge(temp,temp.i,by="TIME_SHORT", all.x=TRUE)
temp.i[is.na(temp.i)] <- 0
```

Fourth, we combine these counts to construct a new data frame, which we’ll use as the basis for the stacked bar plot in the first part of Figure 2.

```
stackb <- c()
stackb$W <- temp.w$n
stackb$A <- temp.a$n
stackb$I <- temp.i$n
stackb <- as.data.frame(stackb, row.names=temp$TIME_SHORT)
```

In addition, we read in a second dataset that lists the most common source in every 10 minute block for every day, which is usually only a single device (computed outside of R with a perl script).

```
ttd <- read.table("TRUMP_3D_SHORT.txt", header = TRUE, sep=",")
```

Finally, we set a colour palette based on the most fequent device for each day/timeblock pair in this dataset, which we’ll use to generate the scatterplot in the second part of Figure 2.

```
colour <- c()
colour[ttd$R > ttd$B & ttd$R > ttd$G] <- redc 
colour[ttd$B > ttd$R & ttd$B > ttd$G] <- bluec
colour[ttd$G > ttd$R & ttd$G > ttd$B] <- yellowc
```

## 3.5 Generating Figure 2

We generate the second figure for the paper, which consists of two plots aligned by time of day. The first plots frequency and source by time of day and the second plots most common source by time of day and date.

```
tiff("FIG/Fig2.tiff", width=2700, height=2400, res=250)

  par(mfrow=c(2,1),
    oma=c(3, 0, 3, 0),
    mar=c(1, 4, 0, 2),
    family="avenir")

# Panel 1: Posting Frequency and Device by Time of Day

  barplot(t(stackb),
          border=NA,
          col=c(bluec,redc,yellowc),
          axes=FALSE,
          axisnames = FALSE,
          space=0,
          ylab="Tweet Count")
  box()
  legend("topleft", 
         legend = c("Web", "Android", "iPhone"), 
         col = c(bluec,redc,yellowc), 
         text.col = c(bluec,redc,yellowc), 
         bty="n",
         cex=1)
  axis(side=2, tick = TRUE)
  mtext(text=expression(bold(
    "Change in Device Use on the @realDonaldTrump Twitter Account")), 
        side=3, line=1, las=1, cex=1.5)

# Panel 2: Device by Time of Day and Date

  plot(as.POSIXct(ttd$TIME, format="%H:%M"),as.Date(ttd$DATE), 
       col=adjustcolor(colour, alpha.f =.75),
       pch=18, cex=.75,
       ylab="Date",
       xaxt="n")
  axis(side=1, tick = TRUE, 
       at=c(as.POSIXct("00:00", format="%H:%M"),
            as.POSIXct("03:00", format="%H:%M"),
            as.POSIXct("06:00", format="%H:%M"),
            as.POSIXct("09:00", format="%H:%M"),
            as.POSIXct("12:00", format="%H:%M"),
            as.POSIXct("15:00", format="%H:%M"),
            as.POSIXct("18:00", format="%H:%M"),
            as.POSIXct("21:00", format="%H:%M"),
            as.POSIXct("24:00", format="%H:%M")),
       labels=c("midnight", "3am", "6am", 
                "9 am", "noon", "3pm", "6pm", "9pm", "midnight"))
  axis(side=3, tick = TRUE, labels = FALSE, 
       line =.5, col = NA, col.ticks = 1,
       at=c(as.POSIXct("00:00", format="%H:%M"),
            as.POSIXct("03:00", format="%H:%M"),
            as.POSIXct("06:00", format="%H:%M"),
            as.POSIXct("09:00", format="%H:%M"),
            as.POSIXct("12:00", format="%H:%M"),
            as.POSIXct("15:00", format="%H:%M"),
            as.POSIXct("18:00", format="%H:%M"),
            as.POSIXct("21:00", format="%H:%M"),
            as.POSIXct("24:00", format="%H:%M")))
  mtext(text="Time of Day", side=1, line=3, las=1)  

dev.off()
```

```
## quartz_off_screen 
##                 2
```

Figure 2

# 4 Multiple Correspondence Analysis

## 4.1 Main Analysis

Now we conduct the multiple correspondence analysis (MCA) for the paper.

First, we extract the linguistic data from the dataset – the 63 categorical linguistic variables plus tweet length in words.

```
tweets_mat<-tweets[,2:65]
```

We then run the MCA to extract 5 dimensions based on this dataset, but setting tweet length (in column 1) as a supplementary quantitative variable. This means tweet length is not taken into consideration when we build the MCA dimensions, but we can still assess the degree to which it is correlated with each dimension.

```
mcares <- MCA(tweets_mat, ncp=5, quanti.sup=1)
```

We also inspect the relationship between tweet length and the dimensions, which shows that Dimension 1 has a strong correlation with tweet length, while the other dimensions do not. This means that the effect of texts length has effectively been isolated on Dimension 1 (see Figure 6 below).

```
mcares$quanti.sup
```

```
## $coord
##               Dim 1      Dim 2      Dim 3       Dim 4     Dim 5
## WORDCOUNT 0.8654755 -0.1226605 0.05684362 -0.05895059 0.1153646
```

Finally, we save select components of the MCA output for further analysis.

```
eig <- as.data.frame(mcares$eig)
coordind <- as.data.frame(mcares$ind$coord)
coordvar <- as.data.frame(mcares$var$coord)
contribind <- as.data.frame(mcares$ind$contrib)
contribvar <- as.data.frame(mcares$var$contrib)
```

And we output the main results to text files for manual linguistic interpretation.

```
summary(mcares, nbelements = Inf, nbind=Inf, ncp=5, 
        file = "OUTPUT/MCA_SUMMARY.txt")
summary(mcares, nbelements = Inf, nbind=0, ncp=5, 
        file = "OUTPUT/FEATURE_SUMMARY.txt")
summary(mcares, nbind = Inf, ncp= 5, 
        file = "OUTPUT/TEXT_SUMMARY.txt")
write.table(coordind, "OUTPUT/TEXT_COORDIANTES.txt")
write.table(coordvar, "OUTPUT/FEATURE_COORDIANTES.txt")
write.table(contribind, "OUTPUT/TEXT_CONTRIBUTIONS.txt")
write.table(contribvar, "OUTPUT/FEATURE_CONTRIBUTIONS.txt")
```

## 4.2 Variance Explained

We chose to focus the first 5 dimensions based on interpretability, which we think is by far and away the most important criteria when selecting the number of dimensions to analyse in exploratory multivariate analysis. But we can also look at the eigen values and the amount of variance explained by the dimensions individually and cumulatively.

```
eig
```

There is a bit of a drop in variance explained after the fifth dimension, but not such a clear break. Notably, there isn’t much variance explained overall by normal standards either, but relatively low variance explained is common for MCA due to the focus on categorical data. There are, however, adjustments we can apply to estimate more meaningful variance explained values (see Le Roux & Rouanet 2010).

First, we can compute *pseudo-eigenvalues* based on the mean eigenvalue across all dimension and the number of variables (63) in our full analysis.

```
mean_eig <- mean(eig$eigenvalue)
Q <- nrow(eig)
eig$pseudo <- (Q / (Q - 1)) ^ 2 * (eig$eigenvalue - mean_eig) ^ 2
```

We can then compute adjusted percentages of variance explained based on these pseudo-eigenvalues by dividing these values by the sum of all pseudo-eigenvalues.

```
pseudo_sum <- sum(eig$pseudo)
eig$pseudo_percent <- round(100 * eig$pseudo / pseudo_sum, 2)
```

Finally, we can output these values, which shows that the first five dimensions account for almost 80% of variance, with something of a drop in variance explained between Dimensions 5 and 6, for whatever that’s worth.

```
eig
```

```
sum(eig$pseudo_percent[1:5])
```

```
## [1] 78.61
```

## 4.3 Generating Figure 3

We generate the third figure for the paper, which is a heat map that displays the strength and direction of the relationship between the 63 linguistic features and the four stylistic dimension.

First, we specify the row labels for the heat map.

```
names <-c("Amplifier", "Analytic Neg.", "Attributive Adj.", "Auxiliary Do", 
          "Copula Be", "Parenthese", "Capital", "Coordinating Conj.", 
          "Contrastive Conj.", "Colon", "Comma", "Definite Art.", 
          "Quantitative Det.", "Exclamation Mark", "First Pers. Pron.", 
          "Period", "Gerund", "Hashtag", "Have Main Verb", "Imperative", 
          "Indefinite Art.", "Infinitive", "Pronoun It", "Modal of Necessity", 
          "Modal of Possibility", "Modal of Prediction", "Non-Initial Ment.", 
          "Phrasal Verb", "Nominalisation", "Numeral Det.", "Numeral NP Head", 
          "Object Pronoun", "Adverb", "Interjection", "Noun", "Verb", "Passive", 
          "Past Tense", "Perception Verb", "Perfect", "Poss. Prop. Noun", 
          "Possessive Det.", "Predicative Adjective", "Preposition", 
          "Aux. Contraction", "Progressive", "Quantitative Pron.", "Proverb Do", 
          "Proper Noun", "Private Verb", "Public Verb", "Question", 
          "Rel. Cl. (Subj. Gap)", "Third Pers. -s", "Second Pers. Pron.", 
          "Stance Verb", "Subject Pronoun", "Superlative", "Third Pers. Pron.", 
          "Time Adverb", "URL", "WH Word", "Initial Mention")
```

Second, we extract the coordinates for just the presence features, which represents the association between the presence of each linguistic feature and each dimension.

```
temp <-coordvar[1:5]
temp
```

```
temp$name <- row.names(temp)
temp<-filter(temp, !grepl("_A",name))
row.names(temp) <- temp$name
temp <-temp[1:5]
coords <- as.matrix(temp)
coords
```

```
##                         Dim 1        Dim 2        Dim 3        Dim 4
## AMPLIFIER_P        0.58361938  0.502930922 -0.080920175 -0.616007278
## ANALNEG_P          0.74486693  0.700595374  0.050076615  1.066730769
## ATTRIBADJ_P        0.28397863 -0.058991986 -0.130876518 -0.156275541
## AUXDO_P            0.79800322  0.974898943  0.212474664  1.888150641
## BEMV_P             0.54348955  0.310607922 -0.364334924 -0.282355229
## BRACKET_P          0.67117862 -0.087809874  0.057281556  0.202150471
## CAPS_P             0.21829449 -0.578012837  0.505655492 -0.177447985
## CCONJ_P            0.43125321  0.002592819  0.056294918 -0.211774230
## CNTRSTCONJ_P       0.82099325  0.728140108  0.023177657  0.179401261
## COLON_P           -0.32204122 -1.142825590  0.042818666  1.092784130
## COMMA_P            0.39103860  0.024524188  0.186898652 -0.163039554
## DEFART_P           0.47498883 -0.173556530 -0.166026898 -0.079031948
## DETQUAN_P          0.65254469  0.197922586 -0.119568599 -0.335696160
## EXCLAM_P           0.04913780  0.155006928  0.443273643 -0.433245442
## FSTPP_P            0.51396408  0.046015888  0.706561738 -0.118990667
## FULSTOP_P          0.25655972  0.186214399 -0.095565021 -0.111849499
## GERUND_P           0.44554968 -0.252626698 -0.174316691 -0.089975635
## HASHTAG_P         -0.58488858 -0.613076035  0.864939599  0.135427871
## HAVEMV_P           0.64170600  0.312932652 -0.033816218 -0.020539578
## IMPERATIVE_P       0.11935479 -0.148614484  0.757424794  0.358803167
## INDEFART_P         0.50061165  0.046880054 -0.314431124 -0.149313245
## INFINITIVE_P       0.64337390  0.088084263  0.190280864  0.298044891
## IT_P               0.67652956  0.484603722 -0.140360143  0.078236937
## MDNEC_P            0.61361641  0.065469313 -0.221042313 -0.170091207
## MDPOSS_P           0.62972161  0.542258865 -0.036001708  0.903601745
## MDPRED_P           0.46687878  0.112455675  0.561975105 -0.258174236
## MENTION_P          0.05966632 -0.475081873 -0.106276626  0.174978157
## MULTIWVB_P         0.51066656  0.036712386  0.376251276  0.138295879
## NOMIN_P            0.37772611 -0.276286842 -0.315697018  0.022600977
## NUMDET_P           0.28261578 -0.660721454  0.084561763 -0.214262174
## NUMNOUN_P          0.35422187 -0.551005376  0.122430549 -0.094492918
## OBJPRO_P           0.74740871  0.170566942  0.856085948  0.196313297
## OTHERADV_P         0.51177420  0.008592735 -0.055755744 -0.002167273
## OTHRINTJ_P        -1.19193549  0.698021979  0.459451661 -0.418737972
## OTHRNOUN_P         0.27393034 -0.129640607 -0.065977233  0.009353581
## OTHRVERB_P         0.40067011 -0.122006860  0.067077599  0.054261159
## PASSIVE_P          0.75877278  0.001210963 -0.138064632 -0.239209992
## PAST_P             0.60332343 -0.034268964 -0.047088685 -0.133029702
## PERCEPTVB_P        0.35128202 -0.012655255  0.699425421  0.080749753
## PERFECT_P          0.76574164  0.100273842 -0.185399968 -0.353287378
## POSESPRPN_P        0.13879929 -0.995164824 -0.614644132  0.222588763
## POSSDET_P          0.52909518  0.039289234  0.372967786  0.021772268
## PREDADJ_P          0.56955164  0.588346866 -0.366387903 -0.434308077
## PREP_P             0.31982180 -0.247335856 -0.008096495 -0.017571585
## PROCONTRACT_P      0.63889889  0.680304718  0.348773041  0.312121758
## PROGRESSIVE_P      0.65966734  0.092278459  0.261277394 -0.126195071
## PROQUAN_P          0.62891323  0.412294652  0.180789679 -0.020999863
## PROVDO_P           0.71673036  0.646574857  0.227604691  0.303573594
## PRPN_P             0.18822573 -0.267401255  0.011634067 -0.053062877
## PRVV_P             0.61851314  0.591646994  0.094767899  0.947149459
## PUBV_P             0.60361746  0.057275814  0.179831615  0.229833030
## QUES_P             0.48783922  0.212902638 -0.421211676  1.552291086
## RELCLAUSESUBGAP_P  0.82378791  0.154268924 -0.292751348 -0.110705141
## SINFLECT_P         0.54198366  0.033928728 -0.472744663 -0.028004741
## SNDPP_P           -0.27801558  0.457048175  0.541045507  0.290466563
## STANCEVB_P         0.64026832  0.490775690  0.435819127  0.294258851
## SUBJPRO_P          0.68049333  0.338200614  0.475922414 -0.014822034
## SUPERLATIVE_P      0.41150406 -0.341210320 -0.763127798 -0.380215808
## THRDPP_P           0.82082790  0.387542061  0.042858612  0.206493414
## TIMEADV_P          0.32841540 -0.282492141  0.703930263 -0.492439064
## URL_P             -0.41582095 -0.893435224  0.214968501  0.355039948
## WHW_P              0.65079231  0.374827568 -0.229858976  1.141909038
## INITIALMENTION_P  -1.42570337  1.334630535 -0.219016072 -0.338650855
##                          Dim 5
## AMPLIFIER_P       -0.075589817
## ANALNEG_P          0.181832590
## ATTRIBADJ_P        0.099721692
## AUXDO_P            0.549426741
## BEMV_P             0.449565542
## BRACKET_P         -0.309230586
## CAPS_P             0.203660753
## CCONJ_P            0.215045550
## CNTRSTCONJ_P       0.029698844
## COLON_P            0.535543214
## COMMA_P            0.244479396
## DEFART_P           0.098510160
## DETQUAN_P          0.185769769
## EXCLAM_P           0.126719497
## FSTPP_P           -0.203629150
## FULSTOP_P          0.076285735
## GERUND_P           0.109013521
## HASHTAG_P          0.346996632
## HAVEMV_P          -0.133521475
## IMPERATIVE_P       0.967850261
## INDEFART_P         0.143354459
## INFINITIVE_P       0.053278582
## IT_P               0.619830549
## MDNEC_P           -0.679431285
## MDPOSS_P           0.455511142
## MDPRED_P           0.019825474
## MENTION_P         -0.116494542
## MULTIWVB_P         0.115858732
## NOMIN_P            0.248697360
## NUMDET_P          -0.102447727
## NUMNOUN_P         -0.144728284
## OBJPRO_P          -0.679360047
## OTHERADV_P        -0.024126290
## OTHRINTJ_P         0.185206856
## OTHRNOUN_P         0.061060764
## OTHRVERB_P        -0.041924259
## PASSIVE_P         -0.810247718
## PAST_P            -0.467757545
## PERCEPTVB_P        0.596861091
## PERFECT_P         -0.421952214
## POSESPRPN_P       -0.259368909
## POSSDET_P          0.089042718
## PREDADJ_P          0.596359098
## PREP_P             0.058205516
## PROCONTRACT_P      0.703328659
## PROGRESSIVE_P     -0.264552646
## PROQUAN_P          0.249317019
## PROVDO_P          -0.119864857
## PRPN_P            -0.045013099
## PRVV_P             0.505733604
## PUBV_P            -0.667667862
## QUES_P            -0.608776943
## RELCLAUSESUBGAP_P -0.193353246
## SINFLECT_P         0.005250457
## SNDPP_P            1.026991669
## STANCEVB_P         0.103250877
## SUBJPRO_P         -0.369787646
## SUPERLATIVE_P      0.710055121
## THRDPP_P          -0.702271104
## TIMEADV_P          0.137151796
## URL_P              0.066156518
## WHW_P             -0.339918135
## INITIALMENTION_P  -0.462048615
```

Third, we isolate the four main stylistic dimensions (Dimension 2-5). We exclude Dimension 1 from our main stylistic analysis because it essentially represents tweet length. We especially don’t want strong Dimension 1 scores to dominate the heat map.

```
adjust <- as.data.frame(coords[,2:5])
adjust <- as.matrix(adjust)
```

Fourth, we set a colour palette.

```
pal <- colorRampPalette(c(bluec,"white",redc), bias=1)(20)
```

Finally, we generate a heat map to show which linguistic features are most strongly associated with the positive (red) and negative (blue) pole of each dimension, with rows organised based on a complete-linkage hierachical cluster analysis. Note the style labels on the four dimensions represent our linguistic interpretations of the MCA results and are discussed at length in the paper.

```
tiff("FIG/Fig3.tiff", width=2700, height=2400, res=400)
  par(family="avenir")
  heatmap(adjust, 
          Colv=NA, 
          cexRow=.5, cexCol=.85, 
          col=pal, las=2, 
          labRow=names,
          labCol=c("Dimension 2:\nConversational\nStyle", 
                   "Dimension 3:\nCampaigning\nStyle",
                   "Dimension 4:\nEngaged\nStyle", 
                   "Dimension 5:\nAdvisory\nStyle"),
          main=" ",
          cex=.5)
  mtext(text=expression(bold(
    "Linguistic Feature Coordinates across 4 Stylistic Dimensions")), 
        side=3, line=2.5, las=1, cex=1)
dev.off()
```

```
## quartz_off_screen 
##                 2
```

Figure 3

## 4.4 Additional Feature Graphs

In addition to the heat map, which just shows coordinates, we also generate plots to let us look at the coordinates and contributions together for each variable on each dimension. These graphs aren’t included in the paper.

We extract the presence contributions like we did for the coordinates.

```
temp <-contribvar[1:5]
temp$name <- row.names(temp)
temp<-filter(temp, !grepl("_A",name))
row.names(temp) <- temp$name
temp <-temp[1:5]
contribs <- as.matrix(temp)
contribs
```

```
##                        Dim 1        Dim 2       Dim 3        Dim 4
## AMPLIFIER_P       0.87865370 1.326342e+00 0.045685434 2.840187e+00
## ANALNEG_P         1.56742038 2.818663e+00 0.019160367 9.327272e+00
## ATTRIBADJ_P       1.00434777 8.810071e-02 0.576955141 8.824939e-01
## AUXDO_P           0.72185910 2.189994e+00 0.138408653 1.172555e+01
## BEMV_P            1.95735265 1.299548e+00 2.378998048 1.532834e+00
## BRACKET_P         0.48240872 1.678442e-02 0.009503298 1.269712e-01
## CAPS_P            0.21640368 3.084144e+00 3.140467729 4.148949e-01
## CCONJ_P           1.23338421 9.062720e-05 0.056843116 8.629723e-01
## CNTRSTCONJ_P      0.69799980 1.116058e+00 0.001504600 9.670391e-02
## COLON_P           0.16636271 4.258666e+00 0.007954358 5.558002e+00
## COMMA_P           1.02110147 8.163935e-03 0.630881479 5.150300e-01
## DEFART_P          1.93901363 5.262312e-01 0.640732544 1.557530e-01
## DETQUAN_P         0.61576152 1.151498e-01 0.055915485 4.728264e-01
## EXCLAM_P          0.01917391 3.878485e-01 4.220159873 4.324782e+00
## FSTPP_P           1.91603785 3.122013e-02 9.793650024 2.979760e-01
## FULSTOP_P         0.82052113 8.786581e-01 0.307904852 4.524784e-01
## GERUND_P          0.21398634 1.398405e-01 0.088588537 2.531982e-02
## HASHTAG_P         0.79096980 1.766538e+00 4.678331353 1.230401e-01
## HAVEMV_P          0.52058702 2.516538e-01 0.003910002 1.547466e-03
## IMPERATIVE_P      0.03567937 1.124450e-01 3.886174426 9.355498e-01
## INDEFART_P        1.33965872 2.388081e-02 1.429384681 3.457848e-01
## INFINITIVE_P      1.40033033 5.335571e-02 0.331283379 8.719346e-01
## IT_P              1.08612782 1.132821e+00 0.126444745 4.214519e-02
## MDNEC_P           0.44542732 1.030716e-02 0.156329000 9.930327e-02
## MDPOSS_P          0.49992346 7.535295e-01 0.004419349 2.986602e+00
## MDPRED_P          0.78841041 9.297955e-02 3.089470390 6.994985e-01
## MENTION_P         0.02037885 2.626266e+00 0.174864818 5.085173e-01
## MULTIWVB_P        0.49130069 5.161529e-03 0.721330502 1.045461e-01
## NOMIN_P           0.58231237 6.332906e-01 1.100142067 6.048858e-03
## NUMDET_P          0.18227684 2.025145e+00 0.044135955 3.039811e-01
## NUMNOUN_P         0.27493539 1.352297e+00 0.088831151 5.676692e-02
## OBJPRO_P          1.09877715 1.163225e-01 3.898825898 2.199428e-01
## OTHERADV_P        1.54782054 8.869667e-04 0.049687697 8.053933e-05
## OTHRINTJ_P        3.50437058 2.442999e+00 1.408283115 1.254891e+00
## OTHRNOUN_P        1.21693547 5.540513e-01 0.190933120 4.116803e-03
## OTHRVERB_P        2.08976176 3.938873e-01 0.158410407 1.112034e-01
## PASSIVE_P         0.77614103 4.018456e-06 0.069500485 2.238168e-01
## PAST_P            2.21924415 1.455416e-02 0.036563151 3.130537e-01
## PERCEPTVB_P       0.15142750 3.994987e-04 1.623609354 2.321626e-02
## PERFECT_P         0.87018253 3.033205e-02 0.137965641 5.374264e-01
## POSESPRPN_P       0.02631134 2.749407e+00 1.395473881 1.963323e-01
## POSSDET_P         1.26783606 1.421095e-02 1.703899313 6.229020e-03
## PREDADJ_P         1.06505125 2.310212e+00 1.192044176 1.796872e+00
## PREP_P            1.45836681 1.772986e+00 0.002527845 1.277290e-02
## PROCONTRACT_P     0.50162783 1.156125e+00 0.404304868 3.473620e-01
## PROGRESSIVE_P     1.07107739 4.260417e-02 0.454443986 1.137294e-01
## PROQUAN_P         0.58698540 5.127927e-01 0.131189446 1.898872e-03
## PROVDO_P          0.63401114 1.048826e+00 0.172923298 3.300123e-01
## PRPN_P            0.47460924 1.947088e+00 0.004903963 1.094402e-01
## PRVV_P            0.87743535 1.632011e+00 0.055711592 5.969957e+00
## PUBV_P            0.98589844 1.804396e-02 0.236671785 4.147156e-01
## QUES_P            0.29330331 1.135550e-01 0.591384774 8.616418e+00
## RELCLAUSESUBGAP_P 0.68119180 4.855971e-02 0.232670964 3.569366e-02
## SINFLECT_P        2.30102313 1.833008e-02 4.734865743 1.782496e-02
## SNDPP_P           0.25534078 1.402770e+00 2.615503419 8.087068e-01
## STANCEVB_P        0.60656335 7.244340e-01 0.760099602 3.717304e-01
## SUBJPRO_P         3.19897034 1.606171e+00 4.231950537 4.403469e-03
## SUPERLATIVE_P     0.17371307 2.427779e-01 1.615788803 4.302902e-01
## THRDPP_P          2.85154591 1.292095e+00 0.021026073 5.236070e-01
## TIMEADV_P         0.38097257 5.729807e-01 4.733820030 2.485241e+00
## URL_P             0.99709613 9.356879e+00 0.720741867 2.109093e+00
## WHW_P             0.83418528 5.624985e-01 0.281453351 7.451739e+00
## INITIALMENTION_P  4.73740375 8.438879e+00 0.302370777 7.755384e-01
##                          Dim 5
## AMPLIFIER_P       4.592513e-02
## ANALNEG_P         2.910295e-01
## ATTRIBADJ_P       3.858846e-01
## AUXDO_P           1.066174e+00
## BEMV_P            4.172902e+00
## BRACKET_P         3.190577e-01
## CAPS_P            5.868934e-01
## CCONJ_P           9.555646e-01
## CNTRSTCONJ_P      2.845907e-03
## COLON_P           1.433466e+00
## COMMA_P           1.243597e+00
## DEFART_P          2.598613e-01
## DETQUAN_P         1.554918e-01
## EXCLAM_P          3.973119e-01
## FSTPP_P           9.370956e-01
## FULSTOP_P         2.260294e-01
## GERUND_P          3.991351e-02
## HASHTAG_P         8.674203e-01
## HAVEMV_P          7.022453e-02
## IMPERATIVE_P      7.310031e+00
## INDEFART_P        3.422789e-01
## INFINITIVE_P      2.992088e-02
## IT_P              2.840657e+00
## MDNEC_P           1.701529e+00
## MDPOSS_P          8.150228e-01
## MDPRED_P          4.429520e-03
## MENTION_P         2.420459e-01
## MULTIWVB_P        7.879443e-02
## NOMIN_P           7.865200e-01
## NUMDET_P          7.462919e-02
## NUMNOUN_P         1.430053e-01
## OBJPRO_P          2.828517e+00
## OTHERADV_P        1.071792e-02
## OTHRINTJ_P        2.636235e-01
## OTHRNOUN_P        1.883984e-01
## OTHRVERB_P        7.128844e-02
## PASSIVE_P         2.757517e+00
## PAST_P            4.156346e+00
## PERCEPTVB_P       1.362087e+00
## PERFECT_P         8.232615e-01
## POSESPRPN_P       2.862660e-01
## POSSDET_P         1.118814e-01
## PREDADJ_P         3.638190e+00
## PREP_P            1.505027e-01
## PROCONTRACT_P     1.894085e+00
## PROGRESSIVE_P     5.367358e-01
## PROQUAN_P         2.874188e-01
## PROVDO_P          5.525028e-02
## PRPN_P            8.457100e-02
## PRVV_P            1.827790e+00
## PUBV_P            3.758331e+00
## QUES_P            1.423133e+00
## RELCLAUSESUBGAP_P 1.169249e-01
## SINFLECT_P        6.728346e-04
## SNDPP_P           1.085628e+01
## STANCEVB_P        4.914791e-02
## SUBJPRO_P         2.943288e+00
## SUPERLATIVE_P     1.611513e+00
## THRDPP_P          6.503560e+00
## TIMEADV_P         2.070213e-01
## URL_P             7.863846e-02
## WHW_P             7.090744e-01
## INITIALMENTION_P  1.550325e+00
```

We convert both the feature coordinates and contributions to data frames.

```
coords <-as.data.frame(coords)
contribs <-as.data.frame(contribs)
```

And then we make a series of plots show both the feature coordinates and contributions for each dimensions. Notably, we see they are fairly similar, aside from the fact that contributions are all positive and thus are not associated with a pole, which is why we focus primarily on presenting coordinates in the paper.

```
plot(coords$`Dim 2`,contribs$`Dim 2`, type="n",
       xlab="Coordinates", ylab="Contributions",
       main="Dimension 2: Conversational Style")
text(coords$`Dim 2`,contribs$`Dim 2`,labels=names, 
       cex=.5, col = bluec)
```

```
plot(coords$`Dim 3`,contribs$`Dim 3`, type="n",
       xlab="Coordinates", ylab="Contributions",
       main="Dimension 3: Campaigning Style")
text(coords$`Dim 3`,contribs$`Dim 3`,labels=names, 
       cex=.5, col = redc)
```

```
plot(coords$`Dim 4`,contribs$`Dim 4`, type="n",
       xlab="Coordinates", ylab="Contributions",
       main="Dimension 4: Engaged Style")
text(coords$`Dim 4`,contribs$`Dim 4`,labels=names, 
       cex=.5, col = greenc)
```

```
plot(coords$`Dim 5`,contribs$`Dim 5`, type="n",
       xlab="Coordinates", ylab="Contributions",
       main="Dimension 5: Advisory Style")
text(coords$`Dim 5`,contribs$`Dim 5`,labels=names, 
       cex=.5, col = orangec)
```

Also note that although we focus in the paper and in this markdown on the presence of features, this is for simplicity’s sake; when interpreting the dimensions, we also consider the absence of features, which are assigned their own contributions and coordinates by the MCA, and which show a complementary pattern to the presence of features.

# 5 Visualising Stylistic Change

## 5.1 Second Time Series Graphing Function

Next, we make a second time series graphing function, which we’ll use to plot change over time in the 4 stylistic dimensions from 2008-2019, including moving average trend lines.

```
tsgraph2 <- function(dim, win, lab, colo, miny, maxy){
  ts<-merge(as.Date(tweets$DATE), dim, by="row.names")
  ts<-aggregate(y ~ x, ts, mean)
  sts <- ts
  for (i in 1:ncol(ts)){
    sts[,i] <-  ma(ts[,i], order = win, centre = T)
  } 
  plot(ts$x, ts$y, type="n", 
       ylim=c(miny, maxy), 
       ylab=lab, xlab=" ", 
       axes = FALSE, frame.plot=TRUE)
  axis(side=2, at=c(-.1,0,.1),labels=c("-0.1","0","+0.1"))
  abline(v=as.Date("2010-09-16"), col="grey",lwd=.6) # Apprentice
  abline(v=as.Date("2011-04-30"), col="grey",lwd=.6) # Birther Attacks
  abline(v=as.Date("2012-11-06"), col="grey",lwd=.6) # 2012 Election
  abline(v=as.Date("2015-06-16"), col="grey",lwd=.6) # Announces
  abline(v=as.Date("2016-02-01"), col="grey",lwd=.6) # Iowa
  abline(v=as.Date("2016-05-03"), col="grey",lwd=.6) # Presumptive Nominee
  abline(v=as.Date("2016-11-08"), col="grey",lwd=.6) # Election
  abline(v=as.Date("2017-01-20"), col="grey",lwd=.6) # Inauguration
  abline(v=as.Date("2010-03-14"), col="grey",lwd=.6) # Apprentice 
  abline(v=as.Date("2011-03-06"), col="grey",lwd=.6) # Apprentice 
  abline(v=as.Date("2012-02-19"), col="grey",lwd=.6) # Apprentice 
  abline(v=as.Date("2013-03-03"), col="grey",lwd=.6) # Apprentice 
  abline(v=as.Date("2015-01-04"), col="grey",lwd=.6) # Apprentice 
  abline(a=0, b=0, lty=1, lwd=.5, col="black")
  points(ts$x, ts$y, pch=16, cex=.7, 
         col= adjustcolor(colo, alpha.f = 0.2))
  lines(ts$x, sts$y, col = colo, lwd=1)
}
```

## 5.2 Generating Figure 4

We use this graphing function to generate the fourth figure for the paper, which presents four aligned time series from 2009-2018 together on one grid, one for each set of dimension coordinates, with individual tweets marked as points and with the moving average trend line superimposed. A number of important dates are also marked.

```
tiff("FIG/Fig4.tiff", width=2700, height=4200, res=400)

par(mfrow=c(4,1),
    oma=c(2, 0, 8, 0),
    family="avenir")

# Panel 1: Dimension 2

  par(mar=c(1, 5, 1, 2))
  tsgraph2(coordind$'Dim 2', 60, 
           "Dimension 2: Conversational", 
           bluec, min=-.15, max=+.15)
  axis(side=3, tick = TRUE,  
       las=2, cex.axis=.85, outer=FALSE,
       col.ticks = "gray50", 
       col.axis = "gray50", col=NA,
       at=c(as.Date("2010-03-14"),
            as.Date("2010-09-16"),
            as.Date("2011-03-06"),
            as.Date("2011-04-30"), 
            as.Date("2012-02-19"),
            as.Date("2012-11-06"), 
            as.Date("2013-03-03"), 
            as.Date("2015-01-04"),
            as.Date("2015-06-16"), 
            as.Date("2016-02-01"), 
            as.Date("2016-05-03"), 
            as.Date("2016-11-08"),
            as.Date("2017-01-20")),
       labels=c("Apprentice", "Apprentice", "Apprentice", 
                "Birther", "Apprentice", "Election",  
                "Apprentice", "Apprentice", "Declares", 
                "Iowa", "Nominee",
                "Election", "Inaguration"))
  mtext(text=expression(bold(
    "Stylistic Change on the @realDonaldTrump Twitter Account")), 
        side=3, line=6, las=1, cex=1)
  axis(side=1, tick = TRUE, cex.axis=1, labels=FALSE,
       at=c(as.Date("2010-01-01"), 
            as.Date("2011-01-01"), 
            as.Date("2012-01-01"),
            as.Date("2013-01-01"), 
            as.Date("2014-01-01"), 
            as.Date("2015-01-01"), 
            as.Date("2016-01-01"), 
            as.Date("2017-01-01"),
            as.Date("2018-01-01")))

# Panel 2: Dimension 3

  par(mar=c(1, 5, 1, 2))
  tsgraph2(coordind$'Dim 3', 60, 
           "Dimension 3: Campaigning", 
           redc, min=-.15, max=+.15)
  axis(side=1, tick = TRUE, cex.axis=1, labels=FALSE,
       at=c(as.Date("2010-01-01"), 
            as.Date("2011-01-01"), 
            as.Date("2012-01-01"),
            as.Date("2013-01-01"), 
            as.Date("2014-01-01"), 
            as.Date("2015-01-01"), 
            as.Date("2016-01-01"), 
            as.Date("2017-01-01"),
            as.Date("2018-01-01")))

# Panel 3: Dimension 4

  par(mar=c(1, 5, 1, 2))
  tsgraph2(coordind$'Dim 4', 60, 
           "Dimension 4: Engaged", 
           greenc, min=-.15, max=+.15)
  axis(side=1, tick = TRUE, cex.axis=1, labels=FALSE,
       at=c(as.Date("2010-01-01"), 
            as.Date("2011-01-01"), 
            as.Date("2012-01-01"),
            as.Date("2013-01-01"), 
            as.Date("2014-01-01"), 
            as.Date("2015-01-01"), 
            as.Date("2016-01-01"), 
            as.Date("2017-01-01"),
            as.Date("2018-01-01")))

# Panel 4: Dimension 5
  
  par(mar=c(1, 5, 1, 2))
  tsgraph2(coordind$'Dim 5', 60, 
           "Dimension 5: Advisory", 
           orangec, min=-.15, max=+.15)
  axis(side=1, tick = TRUE, cex.axis=1, 
       at=c(as.Date("2010-01-01"), 
            as.Date("2011-01-01"), 
            as.Date("2012-01-01"),
            as.Date("2013-01-01"), 
            as.Date("2014-01-01"), 
            as.Date("2015-01-01"), 
            as.Date("2016-01-01"), 
            as.Date("2017-01-01"),
            as.Date("2018-01-01")),
       labels=c("2010", "2011", "2012",
                "2013", "2014", "2015", 
                "2016", "2017", "2018"))
dev.off()
```

```
## quartz_off_screen 
##                 2
```

Figure 4

## 5.3 Third Time Series Graphing Function

In addition, we make a third time series graphing function, which we’ll use to plot change over time in the 4 stylistic dimensions during the campaign, this time with all four trend lines sumperimposed on one graph and no plotting of individual tweets.

```
tsgraph3 <- 
  function(dim, win, lab, colo, minx, maxx, miny, maxy, titlex){
  ts<-merge(as.Date(tweets$DATE), dim, by="row.names")
  ts<-aggregate(y ~ x, ts, mean)
  sts <- ts
  for (i in 1:ncol(ts)){
    sts[,i] <-  ma(ts[,i], order = win, centre = T)
  } 
  par(mar=c(3, 5, 5, 2))
  plot(ts$x, ts$y, type="n", ylim=c(miny, maxy),
       xlim = c(minx, maxx), ylab=lab, xlab=" ", 
       axes = FALSE,frame.plot=TRUE)
  axis(side=1, tick = TRUE, labels=FALSE,
       at=c(as.Date("2010-01-01"), 
            as.Date("2011-01-01"), 
            as.Date("2012-01-01"),
            as.Date("2013-01-01"), 
            as.Date("2014-01-01"), 
            as.Date("2015-01-01"), 
            as.Date("2016-01-01"), 
            as.Date("2017-01-01"),
            as.Date("2018-01-01")))
  axis(side=2, at=c(-.1,0,.1),labels=c("-0.1","0","+0.1"))
  abline(v=as.Date("2010-09-16"), col="grey",lwd=.6) # Apprentice
  abline(v=as.Date("2011-04-30"), col="grey",lwd=.6) # Birther Attacks
  abline(v=as.Date("2012-11-06"), col="grey",lwd=.6) # 2012 Election
  abline(v=as.Date("2015-06-16"), col="grey",lwd=.6) # Announces
  abline(v=as.Date("2016-02-01"), col="grey",lwd=.6) # Iowa
  abline(v=as.Date("2016-05-03"), col="grey",lwd=.6) # Presumptive Nominee
  abline(v=as.Date("2016-08-17"), col="grey",lwd=.6) # Bannon
  abline(v=as.Date("2016-11-08"), col="grey",lwd=.6) # Election
  abline(v=as.Date("2017-01-20"), col="grey",lwd=.6) # Inauguration
  abline(v=as.Date("2017-05-17"), col="grey",lwd=.6) # Mueller
  abline(v=as.Date("2010-03-14"), col="grey",lwd=.6) # Apprentice 
  abline(v=as.Date("2011-03-06"), col="grey",lwd=.6) # Apprentice 
  abline(v=as.Date("2012-02-19"), col="grey",lwd=.6) # Apprentice 
  abline(v=as.Date("2013-03-03"), col="grey",lwd=.6) # Apprentice 
  abline(v=as.Date("2015-01-04"), col="grey",lwd=.6) # Apprentice 
  abline(v=as.Date("2015-08-06"), col="grey",lwd=.6) # GOP Debate 1
  abline(v=as.Date("2016-03-15"), col="grey",lwd=.6) # Super Tuesday 2/Rubio Out
  abline(v=as.Date("2016-06-20"), col="grey",lwd=.6) # Manafort
  abline(v=as.Date("2016-07-15"), col="grey",lwd=.6) # Pence VP
  abline(v=as.Date("2016-10-07"), col="grey",lwd=.6) # Access Hollywood 
  abline(v=as.Date("2015-10-28"), col="grey",lwd=.6) # GOP Debate 3
  abline(a=0, b=0, lty=1, lwd=.5, col="black")
  points(ts$x, ts$y, pch=16, cex=.7, 
         col= adjustcolor(colo, alpha.f = 0.2))
  lines(ts$x, sts$y, col = colo, lwd=1.5)
  axis(side=3, tick = TRUE,  las=2, cex.axis=.85, 
       outer=FALSE, col.ticks = "gray50", 
       col.axis = "gray50", col=NA,
       at=c(as.Date("2010-03-14"),
            as.Date("2010-09-16"),
            as.Date("2011-03-06"),
            as.Date("2011-04-30"), 
            as.Date("2012-02-19"),
            as.Date("2012-11-06"), 
            as.Date("2013-03-03"), 
            as.Date("2015-01-04"),
            as.Date("2015-06-16"), 
            as.Date("2015-08-06"), 
            as.Date("2015-10-28"), 
            as.Date("2016-02-01"), 
            as.Date("2016-03-15"),
            as.Date("2016-05-03"),
            as.Date("2016-06-20"),
            as.Date("2016-07-15"),
            as.Date("2016-08-17"), 
            as.Date("2016-10-07"),
            as.Date("2016-11-08"),
            as.Date("2017-01-20"),
            as.Date("2017-05-17")),
       labels=c("Apprentice", "Apprentice", "Apprentice", 
                "Birther", "Apprentice", "Election",  
                "Apprentice", "Apprentice", "Declares", 
                "GOP Deb 1", "GOP Deb 3", "Iowa", 
                "Sup Tues 2", "Nominee", "Manafort", 
                "Pence VP", "Bannon", "AH Tape", 
                "Election", "Inaguration","Mueller"))
  mtext(text=titlex, 
        side=3, line=6, las=1, cex=1.5,font = 2)
  axis(side=1, tick = TRUE, cex.axis=1, 
       at=c(as.Date("2010-01-01"), 
            as.Date("2011-01-01"), 
            as.Date("2012-01-01"),
            as.Date("2013-01-01"), 
            as.Date("2014-01-01"), 
            as.Date("2015-01-01"), 
            as.Date("2016-01-01"), 
            as.Date("2017-01-01"),
            as.Date("2018-01-01")),
       labels=c("2010", "2011", "2012",
                "2013", "2014", "2015", 
                "2016", "2017", "2018"))
  
}
```

# 6 Generating Figure 5

We use this graphing function to generate the fifth figure for the paper, which presents four aligned time series from 2009-2018 together on one grid, one for each set of dimension coordinates, with individual tweets marked as points and with a 60-day moving average trend line superimposed. A number of important dates are also marked.

```
win <- 60

tiff("FIG/Fig5.tiff", width=2700, height=1800, res=280)
  par(mar=c(3, 5, 8, 2),
      family="avenir")
  
# Get moving averages for Dimension 2

  ts<-merge(as.Date(tweets$DATE), coordind$'Dim 2', by="row.names")
  ts<-aggregate(y ~ x, ts, mean)
  sts <- ts
  for (i in 1:ncol(ts)){
    sts[,i] <-  ma(ts[,i], order = win, centre = T)
  } 
  
# Plot Dimension 2 (with general lables)
  
  plot(ts$x, ts$y, type="n", 
       ylim=c(-.15, .15),
       xlim = c(as.Date("2015-01-01"), as.Date("2018-01-01")), 
       ylab="Dimension Coordinates", xlab=" ", 
       axes = FALSE,frame.plot=TRUE)
  lines(ts$x, sts$y, col = bluec, lwd=1.5)
  
# Get moving averages for Dimension 3 and Plot
  
  ts<-merge(as.Date(tweets$DATE), coordind$'Dim 3', by="row.names")
  ts<-aggregate(y ~ x, ts, mean)
  sts <- ts
  for (i in 1:ncol(ts)){
    sts[,i] <-  ma(ts[,i], order = win, centre = T)
  } 
  lines(ts$x, sts$y, col = redc, lwd=1.5)
  
# Get moving averages for Dimension 4 and Plot
  
  ts<-merge(as.Date(tweets$DATE), coordind$'Dim 4', by="row.names")
  ts<-aggregate(y ~ x, ts, mean)
  sts <- ts
  for (i in 1:ncol(ts)){
    sts[,i] <-  ma(ts[,i], order = win, centre = T)
  } 
  lines(ts$x, sts$y, col = greenc, lwd=1.5)

 # Get moving averages for Dimension 5 and Plot 
  
  ts<-merge(as.Date(tweets$DATE), coordind$'Dim 5', by="row.names")
  ts<-aggregate(y ~ x, ts, mean)
  sts <- ts
  for (i in 1:ncol(ts)){
    sts[,i] <-  ma(ts[,i], order = win, centre = T)
  } 
  lines(ts$x, sts$y, col = orangec, lwd=1.5)
  
# Add Title
  
  mtext(text=expression(bold(
    "Stylistic Change during the 2016 Campaign on the @realDonaldTrump Twitter Account")), 
        side=3, line=6, las=1, cex=1.25)
  
# Add Year Axis (X)
  
  axis(side=1, tick = TRUE, labels=FALSE,
       at=c(as.Date("2010-01-01"), 
            as.Date("2011-01-01"), 
            as.Date("2012-01-01"),
            as.Date("2013-01-01"), 
            as.Date("2014-01-01"), 
            as.Date("2015-01-01"), 
            as.Date("2016-01-01"), 
            as.Date("2017-01-01"),
            as.Date("2018-01-01")))
  
# Add Coordinate Axis (Y)
  
  axis(side=2, at=c(-.1,0,.1),labels=c("-0.1","0","+0.1"))
  
# Add Time Line Splits (X)
  
  abline(v=as.Date("2010-09-16"), col="grey",lwd=.6)    # Apprentice
  abline(v=as.Date("2011-04-30"), col="grey",lwd=.6)    # Birther Attacks
  abline(v=as.Date("2012-11-06"), col="grey",lwd=.6)    # 2012 Election
  abline(v=as.Date("2015-06-16"), col="grey10",lwd=.6)  # Announces
  abline(v=as.Date("2016-02-01"), col="grey",lwd=.6)    # Iowa
  abline(v=as.Date("2016-05-03"), col="grey",lwd=.6)    # Presumptive Nominee
  abline(v=as.Date("2016-08-17"), col="grey",lwd=.6)    # Bannon
  abline(v=as.Date("2016-11-08"), col="grey10",lwd=.6)  # Election
  abline(v=as.Date("2017-01-20"), col="grey10",lwd=.6)  # Inauguration
  abline(v=as.Date("2017-05-17"), col="grey",lwd=.6)    # Mueller
  abline(v=as.Date("2010-03-14"), col="grey",lwd=.6)    # Apprentice 
  abline(v=as.Date("2011-03-06"), col="grey",lwd=.6)    # Apprentice 
  abline(v=as.Date("2012-02-19"), col="grey",lwd=.6)    # Apprentice 
  abline(v=as.Date("2013-03-03"), col="grey",lwd=.6)    # Apprentice 
  abline(v=as.Date("2015-01-04"), col="grey",lwd=.6)    # Apprentice 
  abline(v=as.Date("2015-08-06"), col="grey",lwd=.6)    # GOP Debate 1
  abline(v=as.Date("2016-03-15"), col="grey",lwd=.6)    # Super Tuesday 2/Rubio Out
  abline(v=as.Date("2016-06-20"), col="grey",lwd=.6)    # Manafort
  abline(v=as.Date("2016-07-15"), col="grey",lwd=.6)    # Pence VP
  abline(v=as.Date("2016-10-07"), col="grey",lwd=.6)    # Access Hollywood 
  abline(v=as.Date("2015-10-28"), col="grey",lwd=.6)    # GOP Debate 3
  abline(v=as.Date("2017-02-13"), col="grey",lwd=.6)    # Flynn
  abline(v=as.Date("2017-07-28"), col="grey",lwd=.6)    # Kelly in
  abline(v=as.Date("2017-08-18"), col="grey",lwd=.6)    # Bannon out
  abline(v=as.Date("2017-09-20"), col="grey",lwd=.6)   # Hurricane
  
# Add zero line (Y)  
  
  abline(a=0, b=0, lty=1, lwd=.5, col="black")
 
# Add Time Line Ticks with Labels (X)
  
  axis(side=3, tick = TRUE,  las=2, cex.axis=.85, outer=FALSE,
       col.ticks = "gray50", 
       col.axis = "gray50", col=NA,
       at=c(as.Date("2010-03-14"),
            as.Date("2010-09-16"),
            as.Date("2011-03-06"),
            as.Date("2011-04-30"), 
            as.Date("2012-02-19"),
            as.Date("2012-11-06"), 
            as.Date("2013-03-03"), 
            as.Date("2015-01-04"),
            as.Date("2015-06-16"), 
            as.Date("2015-08-06"), 
            as.Date("2015-10-28"), 
            as.Date("2016-02-01"), 
            as.Date("2016-03-15"),
            as.Date("2016-05-03"),
            as.Date("2016-06-20"),
            as.Date("2016-07-15"),
            as.Date("2016-08-17"), 
            as.Date("2016-10-07"),
            as.Date("2016-11-08"),
            as.Date("2017-01-20"),
            as.Date("2017-02-13"),
            as.Date("2017-05-17"),
            as.Date("2017-07-28"),
            as.Date("2017-08-18"),
            as.Date("2017-09-20")),
       labels=c("Apprentice", "Apprentice", "Apprentice", 
                "Birther", "Apprentice", "Election",  
                "Apprentice", "Apprentice", "Declares", 
                "GOP Deb 1", "GOP Deb 3", "Iowa", 
                "Sup Tue 2", "Nominee", "Manafort", 
                "Pence", "Bannon In", "AH Tape", 
                "Election", "Inaguration", "Flynn",
                "Mueller", "Kelly", "Bannon Out",
                "Hurricane"))
  
  # Add Year Ticks with Labels (X) 
  
  axis(side=1, tick = TRUE, cex.axis=1, 
       at=c(as.Date("2010-01-01"), 
            as.Date("2011-01-01"), 
            as.Date("2012-01-01"),
            as.Date("2013-01-01"), 
            as.Date("2014-01-01"), 
            as.Date("2015-01-01"), 
            as.Date("2016-01-01"), 
            as.Date("2017-01-01"),
            as.Date("2018-01-01")),
       labels=c("2010", "2011", "2012",
                "2013", "2014", "2015", 
                "2016", "2017", "2018"))
  
# Add Legend
  
  legend("topright", 
         legend = c("Conversational", 
                    "Campaigning",
                    "Engaged",
                    "Advisory"), 
         col = c(bluec,redc,greenc,orangec), 
         text.col = c(bluec,redc,greenc,orangec), 
         bty="n",
         cex=1)

dev.off()
```

```
## quartz_off_screen 
##                 2
```

Figure 5

# 7 Graphing Text Length

Finally, we generate the sixth figure for the paper, which plots Dimension 1 against tweet length, showing a very strong correlation (see section 7.1 above), with Tweets sent before and after the length shift highlighted in blue and red.

```
color <- c()
color[as.Date(tweets$DATE) >= 
        as.Date("2017-10-07")] <- redc 
color[as.Date(tweets$DATE) < 
        as.Date("2017-10-07")] <- bluec


tiff("FIG/Fig6.tiff", width=2700, height=2500, res=250)
  par(mar=c(4.5, 5, 6, 2),family="avenir")
  plot(tweets$WORDCOUNT,coordind$`Dim 1`,
       pch="-", cex=3.95, cex.lab = 1.15,
       col=adjustcolor(color, alpha.f =.7),
       ylab="Dimension 1 Coordinate",
       xlab="Total Words")
  mtext(text=expression(bold(
    "Dimension 1 Tweet Coordinate vs Tweet Length")), 
        side=3, line=2, las=1, cex=1.5)
  legend("topleft", 
         legend = c("140 Characters Max", 
                    "280 Characters Max"), 
         col = c(bluec,redc), 
         text.col = c(bluec,redc), 
         bty="n",
         cex=1.15)
dev.off()
```

```
## quartz_off_screen 
##                 2
```

Figure 6

# 8 Conclusion

That is the full quantitative analysis. Please see the PLoS one paper for a description of the manual linguistic analysis carried out for this study, especially the interpretation of the 4 stylistic dimensions.These interpretations are based primarily on an analysis of the functional import of the linguistic features most strongly associated with each of the dimensions, and the tweets most strongly associated with each dimension, which are presented in the supplementary materials for this paper.
